# Synthesis and Antiproliferative Evaluation of Novel Hybrids of Dehydroabietic Acid Bearing 1,2,3-Triazole Moiety

**DOI:** 10.3390/molecules24224191

**Published:** 2019-11-19

**Authors:** Fang-Yao Li, Lin Huang, Qian Li, Xiu Wang, Xian-Li Ma, Cai-Na Jiang, Xiao-Qun Zhou, Wen-Gui Duan, Fu-Hou Lei

**Affiliations:** 1 School of Chemistry and Chemical Engineering, Guangxi University, Nanning 530004, Guangxi, China; lifangyao2006@163.com (F.-Y.L.); wangxiumt2015@163.com (X.W.); 2 College of Pharmacy, Guilin Medical University, Guilin 541100, Guangxi, China; 14795985531@163.com (L.H.); lifangyao@glmc.edu.cn (Q.L.); mxl78@glmc.edu.cn (X.-L.M.); fyli2006@glmc.edu.cn (C.-N.J.); 3 College of Humanities and Management, Guilin Medical University, Guilin 541100, Guangxi, China; zhouxqgy@163.com; 4 Guangxi Key Laboratory of Chemistry and Engineering of Forest Products, Nangning, Guangxi 530006, China; leifuhou@gmail.com

**Keywords:** dehydroabietic acid, 1,2,3-ttriazole, antiproliferative activity, click chemistry

## Abstract

To discover novel potent cytotoxic diterpenoids, a series of hybrids of dehydroabietic acid containing 1,2,3-triazole moiety were designed and synthesized. The target compounds were characterized by means of FT-IR, ^1^H NMR, ^13^C NMR, ESI-MS and elemental analysis techniques. The in vitro cytotoxicity of these compounds was evaluated by standard MTT (methyl thiazolytetrazolium) assay against CNE-2 (nasopharynx), HepG2 (liver), HeLa (epithelial cervical), BEL-7402 (liver) human carcinoma cell lines and human normal liver cell (HL-7702). The screening results revealed that most of the hybrids showed significantly improved cytotoxicity over parent compound DHAA. Among them, [1-(3-fluorobenzyl)-1*H*-1,2,3-triazole-4-yl]dehydroabietic acid methyl ester (**3c**), and [1-(2-nitrobenzyl)-1*H*-1,2,3-triazole-4-yl]dehydroabietic acid methyl ester (**3k**) displayed better antiproliferative activity with IC_50_ (50% inhibitory concentration) values of 5.90 ± 0.41 and 6.25 ± 0.37 µM toward HepG2 cells compared to cisplatin, while they exhibited lower cytotoxicity against HL-7702. Therefore, the 1,2,3-triazole-hybrids could be a promising strategy for the synthesis of antitumor diterpenoids and it also proved the essential role of 1,2,3-triazole moiety of DHAA in the biological activity.

## 1. Introduction

Nowadays, cancer has a serious impact on human health with a high mortality rate [1]. The rapid development of drug resistance and the acute side effects of clinical used anticancer drugs are still the major obstacles to effective chemotherapy [2,3]. Therefore, the discovery and development of new drugs and therapies with high efficacy and low side effects is always the basic mission for medicinal chemists. Natural products have played a dominant role in drug discovery, according to their chemically structural diversity, good biological activities and biocompatibility. There has been increased interest in the search of antitumor drugs from natural sources, and many natural or natural based antitumor drugs such as vinblastine, etoposide and paclitaxel were found and clinically used in recent years [4].

Abietanes are a family of natural tricyclic diterpenoids with interesting pharmacological activity including antitumor, antimicrobial, antiviral, antiulcer, and anti-inflammatory activities [5,6,7,8,9]. Interestingly, dehydroabietic acid (DHAA) and its derivatives have exhibited a broad spectrum of biological activities including antibacterial [10,11,12,13], antifungal [14], antiprotozoal [15], antiviral [16], antiulcer [17], antiherpetic [18] and anti-aging [19]. Especially, a number of DHAA derivatives have been reported in recent years which showed significant antitumor property through DNA binding, apoptosis or oncosis inducing mechanisms [20,21,22]. Hence, a significant amount of attention has been diverted towards the structural modification of dehydroabietic acid to develop new anticancer agents.

On the other hand, 1,2,3-triazole, a privileged building block in the discovery of new anticancer agents, plays a key role in enhancing the cytotoxicity towards the cancer cells because of its improved solubility, cell permeability and pharmacokinetic parameters at the binding site [23,24]. It has been reported that hybridization of 1,2,3-triazole framework other anticancer pharmacophores has the potential to provide novel anticancer candidates [25,26,27,28]. Moreover, some of 1,2,3-triazole containing compounds such as Cefatrizine and Carboxyamidotriazole have already been applied in clinics or under clinical trials for fighting against cancers. Click chemistry is considered to be a nearly perfect synthesizing strategy for reaction between azides and alkynes to afford 1,2,3-triazoles under mild conditions. Therefore, it has been widely applied in many aspects of drug discovery, ranging from the design of lead compounds to tagging of biological systems [29,30].

Considering the above benefits and in continuation of our interest in searching for the pharmacological effects of terpenoid derivatives [31,32,33,34]. We envisioned that the combination of the DHAA framework with 1,2,3-triazole unit may afford the desired high-performance anticancer agents. In this study, a series of novel DHAA-1,2,3-triazole hybrids were synthesized and their cytotoxic activities were assessed in vitro against CNE-2 (Nasopharynx), HepG2 (liver), HeLa (epithelial cervical), and BEL-7402 (liver) human cancer cell lines and HL-7702 normal human liver cell line.

## 2. Results and Discussion

### 2.1. Synthesis and Characterization

**Scheme 1:**
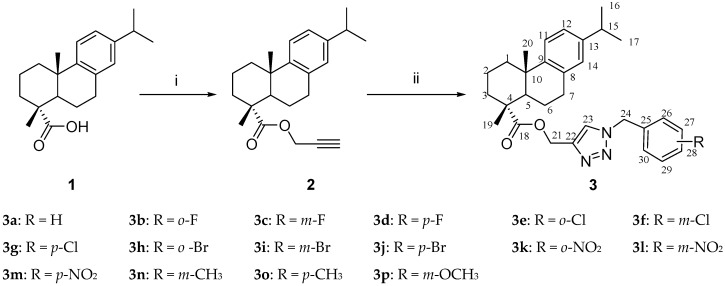
Synthesis of dehydroabietic acid-1,2,3-triazole hybrids. *Reagents and conditions*: (i) propargyl bromide, anhydrous potassium carbonate, DMF, RT, 4 h, yield 86.3%; (ii) benzyl chloride/bromide, NaN_3_, CuI, DMF/H_2_O, RT, 5 h, yield 40.2–60.4%.

DHAA derivatives containing 1,2,3-triazole moiety were synthesized as presented in Scheme 1. All 16 compounds have been confirmed by FT-IR, ^1^H NMR, ^13^C NMR ESI-MS and elemental analysis. As illustrated in Scheme 1, DHAA was treated with propargyl bromide, anhydrous potassium carbonate in dimethylformamide (DMF) to fabricate a good yield of propynyl ester **2.** Huisgen [3+2] cycloaddition of **2** with benzyl chloride/bromide and sodium azide in the presence of cuprous iodide in a DMF aqueous solution resulted in the formation of 1,4 substituted-triazolyl derivatives **3a**–**p** by one-pot route.

The FT-IR spectra of the target compounds **3a**–**p** exhibited characteristic moderate absorption bands at about 1716 cm^−1^ attributed to the stretching vibrations of the C=O. The bands at 1600–1450 cm^−1^ were assigned to the vibration of the skeleton in benzene rings. The bands at 2950–2850 cm^−1^ were assigned to the stretching vibrations of the C-H in methyl or methylene. The ^1^H NMR spectra of the target compounds **3a**–**p** showed characteristic signals at about 7.5 ppm assigned to the triazole protons and signals at 8.30–6.80 ppm assigned to the benzene protons. The ^13^C NMR spectra of the target compounds **3a**–**p** showed peaks for C=O at about 178 ppm, and for aromatic rings at 160–113 ppm.

### 2.2. In Vitro Assay of Antiproliferative Activity

The antiproliferative activities of the 16 library members were tested against four human cancer cell lines including human nasopharyngeal carcinoma cell (CNE-2), human liver cancer cell (HepG2), human hepatocellular carcinoma cell (BEL-7402), human cervical cancer cells (HeLa), and human normal liver cell (HL-7702) using MTT assay according the reported literature method [35]. The tested results were shown in Table 1.

It was found from Table 1 that some of the newly synthesized compounds had significant antineoplastic activity against four tested cancer cell lines, indicating that the introduction of 1,2,3-triazole moiety on the DHAA skeleton increased anti-tumor activity. In particular, compounds **3c** and **3k** exhibited good antitumor activity against HepG2 with IC_50_ values of 5.90 ± 0.41 and 6.25 ± 0.37 μM, better than those of positive control cisplatin. Moreover, compounds **3c** (R = *m*-F), **3d** (R = *p*-F) and **3l** (R = *m*-NO_2_) against CNE-2 with IC_50_ values of 10.92 ± 0.21, 12.20 ± 0.33 and 11.45 ± 0.18 μM, respectively. However, compound **3d** and **3j** showed moderate activity active against BEL-7402 with IC_50_ values of 14.84 ± 075 and 14.53 ± 0.62 μM, respectively. These results declared that introduction of an electron withdrawing group was superior to an electron donating group on the benzene ring against CNE-2 (nasopharynx) cell line. The benzene ring that possessed an electron withdrawing group in *meta*-positions was better than in *ortho*- and *para*- positions. Compound **3b** exhibited the best antitumor activity against Hela (epithelial cervical) with values of 17.76 ± 0.31 μM. Most compounds showed more potent anticancer activities against the HepG2 tumor cells compared to the parent compound. It was important to note that compounds **3a**–**p** showed a certain selectivity against four tumor cell lines and low cytotoxicity on the human normal liver cell (HL-7702).

## 3. Experimental Section

### 3.1. General Information

All commercially available reagents including substituted benzyl chloride/bromide were purchased from Energy Chemicals and used without further purification. Optical rotations were measured on a WZZ-3 polarimeter in CH_2_Cl_2_ at 20 °C (Shanghai Shenguang Instrument, Co., Ltd., Shanghai, China). FT-IR spectra was recorded on a Prestige-21 spectrometer, and samples were prepared as KBr plates (Shimadzu, Co., Ltd., Tokyo, Japan). ^1^H NMR and ^13^C NMR spectra were obtained using a Bruker Avance 600/400 MHz spectrometer in CDCl_3_ with tetramethylsilane (TMS)as an internal standard (Brucker Co., Ltd., Zurich, Switzerland). Elemental analyses were carried out on a PE 2400II elemental analyzer (Perkin Elmer Instruments Co., Ltd., Waltham, MA, USA). Melting points were determined on WRX-4 digital melting point apparatus (uncorrected) (Shanghai YiCe Apparatus & Equipment, Co., Ltd., Shanghai, China). Mass spectra were carried out with a Shimazu liquid chromatograph mass spectrometer (Shimadzu, Co., Ltd., Tokyo, Japan). NMR and IR spectra of compounds **3** can be found in the Supplementary Materials.

### 3.2. Synthesis of Dehydroabietic Acid Propynyl Ester ***2***

In a round bottom flask, anhydrous potassium carbonate (9.4 g) and propargyl bromide (2.8 mL) were added to a solution of compound **1** (9.3 g) in dry DMF (40 mL), and the reaction mixture was stirred at room temperature for 4 h. Reaction was monitored by TLC and the crude product was poured into ice water (200 mL), then the mixture was extracted with ethyl acetate (3 × 20 mL). The combined organic layer was washed with saturated salt water three times, dried over anhydrous sodium sulfate and purified through silica gel chromatography (petroleum ether-EtOAc = 20:1, *v*/*v*) to offer yellow liquid **2** in yield 86.3%; IR (KBr, cm^−1^) 3278 (stretching, m, C≡C-H), 2954, 2933, 2872 (stretching, s, aliphatic C-H), 2127 (stretching, w, C≡C), 1730 (stretching, s, C=O); ^1^H NMR (400 MHz, CDCl_3_): 7.09 (d, 1H, *J* = 8.2 Hz, H-11), 6.92 (dd, 1H, *J* = 8.2, 1.6 Hz, H-12), 6.81 (s, 1H, H-14), 4.59 (ddd, 2H, *J* = 39.7, 15.5, 2.4 Hz, H-22), 2.88 – 2.70 (m, 3H, H-15 and H-7), 2.35 (t, 1H, *J* = 2.4 Hz, H-23), 2.26–2.17 (m, 2H, He-1 and H-5), 1.76 – 1.34 (m, 7H, Ha-1, H-2, H-3 and H-6), 1.22 (s, 3H, H-19), 1.16 (s, 3H, H-20), 1.14 (s, 6H, H-16 and H-17); ^13^C NMR (101 MHz, CDCl_3_) δ 177.70, 146.80, 145.77, 134.71, 126.90, 124.17, 123.94, 77.98, 74.43, 52.00, 47.67, 44.85, 37.96, 36.98, 36.37, 33.47, 30.00, 25.13, 23.96, 21.66, 18.55, 16.48.

### 3.3. General Procedure for the Synthesis of the Target Compounds ***3a**–**p***

A solution of benzyl chloride/bromide (40 mmol) and sodium azide (40 mmol) in *N*,*N*-dimethylformamide (DMF)/water (6 mL, 2:1) was stirred at room temperature. Subsequently, propynyl ester **2** (30 mmol) and CuI (0.002 g) were added, the reaction was performed for 5 h. Upon completion of the reaction, the crude mixture was poured into ice water (20 mL), extracted with CHCl_3_ (3 × 20 mL) and the solution was washed with saturated salt water, dried over Na_2_SO_4_. Purification was performed by chromatography on silica gel (petroleum ether-EtOAc = 5:1, *v*/*v*) to give the target compounds **3a**–**p**, which were characterized by means of FT-IR, ^1^H NMR, ^13^C NMR, ESI-MS and elemental analysis techniques.

*(1-benzyl-1H-1,2,3-triazole-4-yl)dehydroabietic acid methyl ester* (**3a**): White solid; yield 58.0%; m.p. 181.0–183.4 °C; ${\left[\alpha \right]}_{D}^{20}$+26.38 (c 0.012, CH_2_Cl_2_); IR (KBr, cm^−1^) 3126 (stretching, w, C=C-H), 2949, 2868 (stretching, s, aliphatic C-H), 1716 (stretching, s, C=O); ^1^H NMR (600 MHz, CDCl_3_): δ 7.53 (s, 1H, H-23), 7.41 (m, 3H, H-26, H-28 and H-30), 7.30 (m, 2H, H-27 and H-29), 7.17 (d, 1H, *J* = 8.2 Hz, H-11), 7.02 (d, 1H, *J* = 8.1 Hz, H-12), 6.86(s, 1H, H-14), 5.56 (s, 2H, H-24), 5.22 (dd, 2H, *J* = 68.2, 12.7 Hz, H-21), 2.86–2.83 (m, 1H, H-15), 2.78~2.65 (m, 2H, H-7), 2.30 (d, 1H, *J* = 12.6 Hz, He-1), 2.21 (dd,1H, *J* = 12.5 Hz, 1.8, H-5), 1.79–1.46 (m, 7H, Ha-1, H-2, H-3 and H-6), 1.27 (s, 3H, H-19), 1.26 (d, 6H, *J* = 6.9 Hz, H-16 and H-17), 1.21 (s, 3H, H-20); ^13^C NMR (150 MHz, CDCl_3_): δ 178.56, 146.87, 145.83, 143.70, 134.75, 129.30, 128.97, 128.19, 127.02, 124.30, 124.06, 123.67, 57.88, 54.32, 47.69, 44.93, 38.02, 37.04, 36.49, 33.59, 30.05, 25.29, 24.13, 24.12, 21.73, 18.64, 16.59; ESI-MS *m/z*: 470.88 ([M − H]^−^). Anal. Calcd for C_30_H_37_N_3_O_2_: C 76.40, H 7.91, N 8.91; found C 76.39, H 7.93, N 8.88.

*[1-(2-fluorobenzyl)-1H-1,2,3-triazole-4-yl]*dehydroabietic acid methyl ester (**3b**): White solid; yield 46.6%; m.p. 122.4–125.8 °C; ${\left[\alpha \right]}_{D}^{20}$+13.82 (c 0.016, CH_2_Cl_2_); IR (KBr, cm^−1^) 2943, 2866 (stretching, s, aliphatic C-H), 1718 (stretching, s, C=O); ^1^H NMR (600 MHz, CDCl_3_): δ 7.63 (s, 1H, H-23), 7.40 (dd, 1H, *J* = 14.2, 6.8 Hz, H-27), 7.30 (t, 1H, *J* = 7.0 Hz, H-28), 7.21–7.13 (m, 3H, H-29, H-30, H-11), 7.02 (d, 1H, *J* = 8.1, H-12), 6.87 (s, 1H, H-14), 5.62 (s, 2H, H-24), 5.23 (dd, 2H, *J* = 68.3, 12.7 Hz, H-21), 2.85 (m, 1H, H-15), 2.79–2.69 (m, 2H, H-7), 2.31 (d, 1H, *J* = 13.1 Hz, He-1), 2.22 (dd, 1H, *J* = 12.4, 1.4 Hz, H-5), 1.90–1.67 (m, 7H, Ha-1, H-2, H-3 and H-6), 1.28 (s, 3H, H-19), 1.26 (d, 6H, *J* = 6.9 Hz, H-16 and H-17), 1.21 (s, 3H, H-20); ^13^C NMR (150 MHz, CDCl_3_): δ 178.54, 160.68, 146.88, 145.82, 143.68, 134.76, 131.13, 130.70, 127.03, 125.01, 124.31, 124.06, 123.86, 121.98, 116.01, 57.84, 47.85, 47.70, 44.94, 38.03, 37.05, 36.50, 33.60, 30.06, 25.30, 24.13, 21.75, 18.66, 16.61; ESI-MS *m/z*: 488.88 ([M − H]^−^). Anal. Calcd for C_30_H_36_FN_3_O_2_: C 73.59, H 7.41, N 8.58; found C 73.56, H 7.39, N 8.59.

*[1-(3-fluorobenzyl)-1H-1,2,3-triazole-4-yl]*dehydroabietic acid methyl ester (**3c**): White solid; yield 58.7%; m.p. 175.7–178.3 °C; ${\left[\alpha \right]}_{D}^{20}$+17.26 (c 0.014, CH_2_Cl_2_); IR (KBr, cm^−1^) 2919 (stretching, s, aliphatic C-H), 1712 (stretching, s, C=O); ^1^H NMR (600 MHz, CDCl_3_): δ 7.58 (s, 1H, H-23), 7.38 (dd, 1H, *J* = 13.8, 8.0 Hz, H-29), 7.18 (d, 1H, *J* = 8.2 Hz, H-11), 7.09 (m,2H, H-26 and H-28), 7.02 (d, 1H, *J* = 8.2 Hz, H-12), 6.98 (d, 1H, *J* = 9.2 Hz, H-30), 6.87 (s, 1H, H-14), 5.56 (s, 2H, H-24), 5.24 (dd, 2H, *J* = 68.0, 12.7 Hz, H-21), 2.89~2.81 (m, 1H, H-15), 2.79–2.66 (m, 2H, H-7), 2.31 (d, 1H, *J* = 12.5 Hz, He-1), 2.22 (dd, 1H, *J* = 12.5 Hz, 1.8, H-5), 1.84–1.46 (m, 7H, Ha-1, H-2, H-3 and H-6), 1.28 (s, 3H, H-19), 1.26 (d, 6H, *J* = 6.9 Hz, H-16 and H-17), 1.21 (s, 3H, H-20); ^13^C NMR(150 MHz, CDCl_3_): δ 178.57, 163.15, 146.86, 145.86, 143.90, 137.08, 134.71, 130.96, 127.03, 124.30, 124.08, 123.82, 123.66, 116.00, 115.11, 58.82, 53.62, 47.70, 44.94, 38.03, 37.04, 36.51, 33.60, 30.05, 25.29, 24.12, 21.75, 18.64, 16.60; ESI-MS *m/z*: 488.95 ([M − H]^−^). Anal. Calcd for C_30_H_36_FN_3_O_2_: C 73.59, H 7.41, N 8.58; found C 73.59, H 7.38, N 8.57.

*[1-(4-fluorobenzyl)-1H-1,2,3-triazole-4-yl]*dehydroabietic acid methyl ester (**3d**): White solid; yield 55.4%; m.p. 183.0–186.3 °C; ${\left[\alpha \right]}_{D}^{20}$+22.92 (c 0.011, CH_2_Cl_2_); IR (KBr, cm^−1^) 2949 (stretching, s, aliphatic C-H), 1716 (stretching, s, C=O); ^1^H NMR (600 MHz, CDCl_3_): δ 7.54(s, 1H, H-23), 7.30 (dd, 2H, *J* = 8.6, 5.2 Hz, H-26 and H-30), 7.18 (d, 1H, *J* = 8.2 Hz, H-11), 7.10 (t, 2H, *J* = 8.6 Hz, H-27 and H-29), 7.03 (d, 1H, *J* = 8.2 Hz, H-12), 6.87 (s, 1H, H-14), 5.53 (s, 2H, H-24), 5.23 (dd, 2H, *J* = 64.4, 12.7 Hz, H-21), 2.88–2.83 (m, 1H, H-15), 2.77–2.66 (m, 2H, H-7), 2.31 (d, 1H, *J* = 12.7 Hz, He-1), 2.21 (dd, 1H, *J* = 12.5 Hz, 1.8 Hz, H-5), 1.91–1.48 (m, 7H, Ha-1, H-2, H-3 and H-6), 1.28 (s, 3H, H-19), 1.26 (d, 6H, *J* = 6.9 Hz, H-16 and H-17), 1.21 (s, 3H, H-20); ^13^C NMR (150 MHz, CDCl_3_): δ 178.56, 163.02, 146.87, 145.89, 143.82, 134.69, 130.57, 130.10, 127.02, 124.31, 124.10, 123.59, 116.31, 57.86, 53.55, 47.70, 44.94, 38.02, 37.04, 36.50, 33.60, 30.06, 25.29, 24.12, 21.73, 18.64, 16.61; ESI-MS *m/z*: 488.81 ([M − H]^−^). Anal. Calcd for C_30_H_36_FN_3_O_2_: C 73.59, H 7.41, N 8.58; found C 73.60, H 7.38, N 8.56.

*[1-(2-chlorobenzyl)-1H-1,2,3-triazole-4-yl]*dehydroabietic acid methyl ester (**3e**): White solid; yield 60.4%; m.p. 80.6–84.3 °C; ${\left[\alpha \right]}_{D}^{20}$+12.69 (c 0.010, CH_2_Cl_2_); IR (KBr, cm^−1^) 2931 (stretching, s, aliphatic C-H)V, 1720 (stretching, s, C=O); ^1^H NMR (600 MHz, CDCl_3_): δ 7.55 (s, 1H, H-23), 7.38–7.31 (m, 2H, H-28 and H-30), 7.27 (s, 1H, H-27), 7.16 (d, 2H, *J* = 8.2 Hz, H-11 and H-29), 7.00 (d, 1H, *J* = 8.1 Hz, H-12), 6.86 (s, 1H, H-14), 5.52 (s, 2H, H-24), 5.23 (dd, 2H, *J* = 64.3, 12.7 Hz, H-21), 2.84 (dt, 1H, *J* = 13.8, 6.9 Hz, H-15), 2.77~2.65 (m, 2H, H-7), 2.29 (d, 1H, *J* = 12.6 Hz, He-1), 2.20 (dd, 1H, *J* = 12.5, 1.7 Hz, H-5), 1.82~1.47 (m, 7H, Ha-1, H-2, H-3 and H-6), 1.27 (s, 3H, H-19), 1.24 (d, 6H, *J* = 6.9 Hz, H-16 and H-17), 1.20 (s, 3H, H-20); ^13^C NMR (150 MHz, CDCl_3_): δ 178.46, 146.73, 145.73, 143.82, 136.48, 135.09, 134.59, 130.46, 129.07, 128.06, 126.91, 126.06, 124.16, 123.94, 123.64, 57.70, 53.46, 47.57, 44.81, 37.90, 36.91, 36.38, 33.46, 29.90, 25.15, 23.99, 21.62, 18.51, 16.47; ESI-MS *m/z*: 529.00 ([M + Na]^+^). Anal. Calcd for C_30_H_36_ClN_3_O_2_: C 71.20, H 7.17, N 8.30; found C 71.16, H 7.15, N 8.29.

*[1-(3-chlorobenzyl)-1H-1,2,3-triazole-4-yl]*dehydroabietic acid methyl ester (**3f**): White solid; yield 52.3%; m.p. 153.2–155.5 °C; ${\left[\alpha \right]}_{D}^{20}$+27.86 (c 0.012, CH_2_Cl_2_); IR (KBr, cm^−1^) 2939 (stretching, s, aliphatic C-H), 1714 (stretching, s, C=O); ^1^H NMR (600 MHz, CDCl_3_): δ 7.61 (s, 1H, H-23), 7.46 (dd, 1H, *J* = 8.0, 1.1 Hz, H-28), 7.34 (td, 1H, *J* = 7.7, 1.7 Hz, H-29), 7.29 (d, 1H, *J* = 9.0 Hz, H-26), 7.21 (dd, *J* = 7.6, 1.5 Hz, 1H, H-30), 7.16 (d, 1H, *J* = 8.2 Hz, H-11), 7.00 (dd, *J* = 8.1, 1.6 Hz, 1H, H-12), 6.86 (d, *J* = 12.8 Hz, 1H, H-14), 5.68 (s, 2H, H-24), 5.23 (dd, 2H, *J* = 68.2, 12.7 Hz, H-21), 2.84 (dt, 1H, *J* = 13.8, 6.9 Hz, H-15), 2.79–2.67 (m, 2H, H-7), 2.29 (d, 1H, *J* = 12.5 Hz, He-1), 2.21 (dd, *J* = 12.5, 1H, 2.0 Hz, H-5), 1.81~1.47 (m, 7H, Ha-1, H-2, H-3 and H-6), 1.27 (s, 3H, H-19), 1.24 (d, 6H, *J* = 6.9 Hz, H-16 and H-17), 1.20 (s, 3H, H-20); ^13^C NMR (150 MHz, CDCl_3_): δ 178.40, 146.75, 145.70, 143.48, 134.61, 133.54, 132.36, 130.37, 130.32, 129.98, 127.62, 126.89, 124.16, 123.93, 123.86, 57.72, 51.45, 47.57, 44.79, 37.91, 36.92, 36.40, 33.46, 29.96, 25.15, 24.00, 23.98, 21.64, 18.53, 16.47; ESI-MS *m/z*: 506.00 ([M]^+^). Anal. Calcd for C_30_H_36_ClN_3_O_2_: C 71.20, H 7.17, N 8.30; found C 71.21, H 7.14, N 8.32.

*[1-(4-chlorobenzyl)-1H-1,2,3-triazole-4-yl]*dehydroabietic acid methyl ester (**3g**): White solid; yield 44.7%; m.p. 176.9–179.2 °C; ${\left[\alpha \right]}_{D}^{20}$+18.67 (c 0.015, CH_2_Cl_2_); IR (KBr, cm^−1^) 2926 (stretching, s, aliphatic C-H), 1718 (stretching, s, C=O); ^1^H NMR (600 MHz, CDCl_3_): δ 7.52 (s, 1H, H-23), 7.37 (d, 2H, *J* = 8.4 Hz, H-27 and H-29), 7.23 (d, 2H, *J* = 8.4 Hz, H-26 and H-30), 7.17 (s, 1H, H-11), 7.01 (dd, 1H, *J* = 8.1, 1.4 Hz, H-12), 6.86 (s, 1H, H-14), 5.51 (s, 2H, H-24), 5.21 (dd, 2H, *J* = 61.7, 12.7 Hz, H-21), 2.84 (dt, 1H, *J* = 13.8, 6.9 Hz, H-15), 2.77–2.63 (m, 2H, H-7), 2.29 (d, 1H, *J* = 12.9 Hz, He-1), 2.19 (dd, 1H, *J* = 12.5, 1.9 Hz, H-5), 1.81~1.47 (m, 7H, Ha-1, H-2, H-3 and H-6), 1.26 (s, 3H, H-19), 1.24 (d, 6H, *J* = 6.9 Hz, H-16 and H-17), 1.20 (s, 3H, H-20); ^13^C NMR (150 MHz, CDCl_3_): δ 178.44, 146.73, 145.77, 143.77, 134.92, 134.55, 133.04, 129.38, 126.90, 124.16, 123.95, 123.49, 77.25, 77.04, 76.83, 57.72, 53.43, 47.58, 44.81, 37.89, 36.91, 36.39, 33.47, 29.91, 25.15, 23.99, 21.61, 18.51, 16.47; ESI-MS *m/z*: 505.20 ([M − H]^−^). Anal. Calcd for C_30_H_36_ClN_3_O_2_: C 71.20, H 7.17, N 8.30; found C 71.19, H 7.18, N 8.27.

*[1-(2-bromobenzyl)-1H-1,2,3-triazole-4-yl]*dehydroabietic acid methyl ester (**3h**): White solid; yield 48.6%; m.p. 95.1–97.3 °C; ${\left[\alpha \right]}_{D}^{20}$+32.13 (c 0.014, CH_2_Cl_2_); IR (KBr, cm^−1^) 2929 (stretching, s, aliphatic C-H), 1714 (stretching, s, C=O); ^1^H NMR (600 MHz, CDCl_3_): δ 7.67–7.61 (m, 2H, H-23 and H-27), 7.33 (t, 1H, *J* = 7.5 Hz, H-29), 7.26 (t, 1H, *J* = 7.7 Hz, H-28), 7.19–7.14 (m, 2H, H-11 and H-30), 7.00 (d, 1H, *J* = 8.1 Hz, H-12), 6.85 (s, 1H, H-14), 5.68 (s, 2H, H-24), 5.23 (dd, 2H, *J* = 69.0, 12.7 Hz, H-21), 2.83 (dt, 1H, *J* = 13.8, 6.9 Hz, H-15), 2.79–2.68 (m, 2H, H-7), 2.29 (d, 1H, *J* = 12.5 Hz, He-1), 2.21 (dd, 1H, *J* = 12.5, 1.9 Hz, H-5), 1.80–1.45 (m, 7H, Ha-1, H-2, H-3 and H-6), 1.27 (s, 3H, H-19), 1.24 (d, 6H, *J* = 6.9 Hz, H-16 and H-17), 1.20 (s, 3H, H-20); ^13^C NMR (150 MHz, CDCl_3_): δ 178.39, 146.75, 145.70, 143.48, 134.61, 134.06, 133.28, 130.49, 130.39, 128.24, 126.89, 124.17, 123.93, 123.89, 123.52, 57.73, 53.85, 47.58, 44.79, 37.91, 36.92, 36.42, 33.46, 29.98, 25.16, 24.00, 23.99, 21.65, 18.53, 16.48; ESI-MS *m/z*: 550.10 ([M]^+^). Anal. Calcd for C_30_H_36_BrN_3_O_2_: C 65.45, H 6.59, N 7.63; found C 65.49, H 6.63, N 7.60.

*[1-(3-bromobenzyl)-1H-1,2,3-triazole-4-yl]dehydroabietic acid methyl ester* (**3i**): White solid; yield 54.5%; m.p. 146.1–148.0 °C; ${\left[\alpha \right]}_{D}^{20}$+24.59 (c 0.010, CH_2_Cl_2_); IR (KBr, cm^−1^) 2945 (stretching, s, aliphatic C-H), 1716 (stretching, s, C=O); ^1^H NMR (600 MHz, CDCl_3_): δ 7.55 (s, 1H, H-23), 7.52 (1H, d, *J* = 7.9 Hz, H-28), 7.44 (s, 1H, H-26), 7.28 (d, 1H, *J* = 9.7 Hz, H-29), 7.21 (d, 1H, *J* = 7.7 Hz, H-30), 7.16 (d, 1H, *J* = 8.2 Hz, H-11), 7.01 (d, 1H, *J* = 8.1 Hz, H-12), 6.86 (s, 1H, H-14), 5.52 (s, 2H, H-24), 5.23 (dd, 2H, *J* = 63.2, 12.7 Hz, H-21), 2.84 (dt, 1H, *J* = 13.8, 6.9 Hz, H-15), 2.77–2.65 (m, 2H, H-7), 2.29 (d, 1H, *J* = 12.7 Hz, He-1), 2.20 (dd, 1H, *J* = 12.5, 1.9 Hz, H-5), 1.86~1.45 (m, 7H, Ha-1, H-2, H-3 and H-6), 1.27 (s, 3H, H-19), 1.24 (d, 6H, *J* = 6.9 Hz, H-16 and H-17), 1.20 (s, 3H, H-20); ^13^C NMR (150 MHz, CDCl_3_): δ 178.46, 146.73, 145.72, 143.82, 136.72, 134.59, 132.01, 130.97, 130.71, 126.91, 126.54, 124.16, 123.94, 123.64, 123.16, 57.70, 53.39, 47.58, 44.82, 37.90, 36.92, 36.39, 33.47, 29.90, 25.15, 23.99, 21.63, 18.52, 16.47; ESI-MS *m/z*: 572.30 ([M + Na]^+^). Anal. Calcd for C_30_H_36_BrN_3_O_2_: C 65.45, H 6.59, N 7.63; found C 65.47, H 6.62, N 7.61.

*[1-(4-bromobenzyl)-1H-1,2,3-triazole-4-yl]dehydroabietic acid methyl ester* (**3j**): White solid; yield 40.2%; m.p. 172.5–174 °C; ${\left[\alpha \right]}_{D}^{20}$+27.19 (c 0.012, CH_2_Cl_2_); IR (KBr, cm^−1^) 2924 (stretching, s, aliphatic C-H), 1718 (stretching, s, C=O); ^1^H NMR (600 MHz, CDCl_3_): δ 7.56–7.50 (m, 3H, H-23, H-27 and H-29), 7.16 (d, 3H, *J* = 8.2 Hz, H-11, H-26 and H-30), 7.01 (d, 1H, *J* = 9.2 Hz, H-12), 6.86 (s, 1H, H-14), 5.50 (s, 2H, H-24), 5.21 (dd, 2H, *J* = 61.3, 12.7 Hz, H-21), 2.84 (dt, *J* = 13.8, 6.9 Hz, 1H, H-15), 2.76–2.67 (m, 2H, H-7), 2.30 (d, 1H, *J* = 12.9 Hz, He-1), 2.19 (dd, 1H, *J* = 12.5, 1.8 Hz, H-5), 1.80~1.45 (m, 7H, Ha-1, H-2, H-3 and H-6), 1.27 (s, 3H, H-19), 1.25 (d, 6H, *J* = 6.9 Hz, H-16 and H-17), 1.20 (s, 3H, H-20); ^13^C NMR (150 MHz, CDCl_3_): δ 178.44, 146.73, 145.77, 143.78, 134.55, 133.55, 132.35, 129.66, 126.91, 124.16, 123.95, 123.52, 123.03, 57.72, 53.48, 47.58, 44.81, 37.89, 36.92, 36.39, 33.47, 29.91, 25.15, 24.00, 21.61, 18.51, 16.47; ESI-MS *m/z*: 572.10 ([M + Na]^+^). Anal. Calcd for C_30_H_36_BrN_3_O_2_: C 65.45, H 6.59, N 7.63; found C 65.47, H 6.58, N 7.65.

*[1-(2-nitrobenzyl)-1H-1,2,3-triazole-4-yl]dehydroabietic acid methyl ester* (**3k**): White solid; yield 59.8%; m.p. 119.7–122.2 °C; ${\left[\alpha \right]}_{D}^{20}$+28.42 (c 0.016, CH_2_Cl_2_); IR (KBr, cm^−1^) 2929 (stretching, s, aliphatic C-H), 1720 (stretching, s, C=O), 1527 (stretching, s, -NO_2_); ^1^H NMR (600 MHz, CDCl_3_): δ 8.17 (d, 1H, *J* = 7.8 Hz, H-27), 7.76 (1H, s, H-23), 7.61 (t, 1H, *J* = 7.2 Hz, H-29), 7.55 (t, 1H, *J* = 7.5 Hz, H-28), 7.16 (1H, d, *J* = 8.2 Hz, H-11), 7.07 (d, 1H, *J* = 7.7 Hz, H-30), 7.00 (d, 1H, *J* = 8.0 Hz, H-12), 6.85 (1H, s, H-12), 5.95 (s, 2H, H-24), 5.25 (dd, 2H, *J* = 54.6, 12.7 Hz, H-21), 2.82 (1H, m, H-15), 2.77–2.74 (2H, m, H-7), 2.29 (d, 1H, *J* = 12.4 Hz, He-1), 2.22 (dd, 1H, *J =* 12.4, 1.5 Hz, H-5), 1.81–1.44 (m, 7H, Ha-1, H-2, H-3 and H-6), 1.27 (s, 3H, H-19), 1.22 (d, 6H, *J* = 6.9 Hz, H-16 and H-17), 1.20 (s, 3H, H-20); ^13^C NMR (150 MHz, CDCl_3_): δ 178.02, 147.12, 146.41, 145.38, 143.35, 134.24, 134.04, 130.24, 129.98, 129.40, 126.56, 125.10, 124.35, 123.84, 123.62, 57.37, 50.52, 47.26, 44.46, 37.57, 36.58, 36.11, 33.12, 29.65, 24.83, 23.66, 21.34, 18.19, 16.16; ESI-MS *m/z*: 514.90 ([M − H]^−^). Anal. Calcd for C_30_H_36_N_4_O_4_: C 69.74, H 7.02, N 10.84; found C 69.78, H 7.00, N 10.82.

*[1-(3-nitrobenzyl)-1H-1,2,3-triazole-4-yl]dehydroabietic acid methyl ester* (**3l**): White solid; yield 48.3%; m.p. 128.5–132.3 °C; ${\left[\alpha \right]}_{D}^{20}$+6.39 (c 0.011, CH_2_Cl_2_); IR (KBr, cm^−1^) 3074, 2926 (stretching, s, aliphatic C-H), 1712 (stretching, s, C=O), 1533(stretching, s, -NO_2_); ^1^H NMR (600 MHz, CDCl_3_): δ 8.25 (d, 1H, *J* = 7.8 Hz, H-28), 8.19 (s, 1H, H-26), 7.66 (s, 1H, H-23), 7.61 (dt, 2H, *J* = 15.5, 7.7 Hz, H-29 and H-30), 7.17 (d, 1H, *J* = 8.2 Hz, H-11), 7.02 (d, 1H, *J* = 8.2 Hz, H-12), 6.86 (s, 1H, H-14), 5.68 (s, 2H, H-24), 5.24 (dd, 2H, *J* = 47.4, 12.7 Hz, H-21), 2.85 (dt, 1H, *J* = 13.8, 6.9 Hz, H-15), 2.79–2.70 (2H, m, H-7), 2.31 (d, 1H, *J* = 13.0, He-1), 2.21 (dd, 1H, *J* = 12.5, 1.8, H-5), 1.84–1.47(m, 7H, Ha-1, H-2, H-3 and H-6), 1.28 (3H, s, H-19), 1.25 (d, 6H, *J* = 6.9 Hz, H-16 and H-17), 1.21 (3H, s, H-20); ^13^C NMR(150 MHz, CDCl_3_): δ 178.58, 148.70, 146.86, 145.90, 144.15, 136.81, 134.65, 133.98, 130.47, 127.02, 124.30, 124.11, 123.96, 123.94, 122.97, 57.80, 53.23, 47.72, 44.93, 38.02, 37.04, 36.54, 33.59, 30.06, 25.28, 24.12, 18.63, 16.60; ESI-MS *m/z*: 514.90 ([M − H]^−^). Anal. Calcd for C_30_H_36_N_4_O_4_: C 69.74, H 7.02, N 10.84; found C 69.75, H 7.01, N 10.85.

*[1-(4-nitrobenzyl)-1H-1,2,3-triazole-4-yl]dehydroabietic acid methyl* ester (**3m**): White solid; yield 41.1%; m.p. 173.9–176.7 °C; ${\left[\alpha \right]}_{D}^{20}$+16.44 (c 0.012, CH_2_Cl_2_); IR (KBr, cm^−1^) 2949 (stretching, s, aliphatic C-H), 1716 (stretching, s, C=O), 1530(stretching, s, -NO_2_); ^1^H NMR (600 MHz, CDCl_3_): δ 8.26 (d, 2H, *J* = 8.6 Hz, H-27 and H-29), 7.63 (1H, s, H-23), 7.43 (d, 2H, *J* = 8.5 Hz, H-26 and H-30), 7.18 (d, 1H, *J* = 8.2 Hz, H-11), 7.02 (d, 1H, *J* = 8.1Hz, H-12), 6.86 (1H, s, H-14), 5.68 (s, 2H, H-24), 5.24 (dd, 2H, *J* = 48.4, 12.7 Hz, H-21),2.85 (dt, 1H, *J* = 13.8, 6.9 Hz, H-15), 2.80–2.67 (m, 2H, H-7), 2.31 (d, 1H, *J =* 13.1 Hz, He-1), 2.21 (dd, 1H, *J =* 12.4, 1.4 Hz, H-5), 1.84–1.69 (m, 7H, Ha-1, H-2, H-3 and H-6), 1.28 (s, 3H, H-19), 1.25 (d, 6H, *J* = 6.9 Hz, H-16 and H-17), 1.21 (s, 3H, H-20); ^13^C NMR (150 MHz, CDCl_3_): δ 178.58, 148.24, 146.83, 145.99, 144.19, 141.70, 134.59, 128.73, 127.02, 124.47, 124.31,124.14, 124.03, 57.80, 53.24, 47.73, 44.93, 38.01, 37.04, 36.54, 33.58, 30.08, 25.28, 24.11, 21.77, 18.63, 16.61; ESI-MS *m/z*: 514.86 ([M − H]^−^). Anal. Calcd for C_30_H_36_N_4_O_4_: C 69.74, H 7.02, N 10.84; found C 69.75, H 7.04, N 10.81.

*[1-(3-methylbenzyl)-1H-1,2,3-triazole-4-yl]dehydroabietic acid methyl ester* (**3n**): White solid; yield 51.4%; m.p. 130.7–132.8 °C; ${\left[\alpha \right]}_{D}^{20}$+42.99 (c 0.010, CH_2_Cl_2_); IR (KBr, cm^−1^) 2945 (stretching, s, aliphatic C-H), 1716 (stretching, s, C=O); ^1^H NMR (600 MHz, CDCl_3_): δ 7.53 (1H, s, H-23), 7.30 (t, 1H, *J* = 7.6 Hz, H-29), 7.21 (d, 1H, *J* = 7.6 Hz, H-30), 7.17(d, 1H, *J* = 8.2 Hz, H-11), 7.13-.08(m, 2H, H-26 and H-28), 7.02 (d, 1H, *J =* 8.1 Hz, H-12), 6.86 (s, 1H, H-14), 5.51 (s, 2H, H-24), 5.23 (dd, 2H, *J* = 65.3, 12.7 Hz, H-21), 2.85 (dt, 1H, *J* = 13.8, 6.9 Hz, H-15), 2.76–2.67 (m, 2H, H-7), 2.38 (s, 3H, H-31), 2.30 (d, 1H, *J* = 12.6 Hz, He-1), 2.21 (dd, 1H, *J* = 12.4, 1.9 Hz, H-5), 1.80 -1.46 (m, 7H, Ha-1, H-2, H-3 and H-6), 1.28 (s, 3H, H-19), 1.26 (d, 6H, *J* = 6.9 Hz, H-16 and H-17), 1.21 (s, 3H, H-20); ^13^C NMR (150 MHz, CDCl_3_): δ 178.57, 146.88, 145.83, 143.64, 139.12, 134.76, 134.55, 129.71, 129.17, 128.92, 127.02, 125.27, 124.30, 124.06, 123.67, 57.89, 54.33, 47.69, 44.94, 38.03, 37.05, 36.49, 33.60, 30.05, 25.29, 24.13, 21.73, 21.49, 18.65, 16.60; ESI-MS *m/z*: 484.8 ([M − H]^−^). Anal. Calcd for C_31_H_39_N_3_O_2_: C 76.67, H 8.09, N 8.65; found C 76.66, H 8.05, N 8.62.

*[1-(4-methylbenzyl)-1H-1,2,3-triazole-4-yl]dehydroabietic acid methyl ester* (**3o**): White solid; yield 60.1%; m.p. 160.4–162.0 °C; ${\left[\alpha \right]}_{D}^{20}$+28.37 (c 0.013, CH_2_Cl_2_); IR (KBr, cm^−1^) 2927 (stretching, s, aliphatic C-H), 1716 (stretching, s, C=O); ^1^H NMR (600 MHz, CDCl_3_): δ 7.49 (s, 1H, H-23), 7.18 (dt, 5H, *J* = 13.3, 8.2 Hz, H-11, H-26, H-27, H-29 and H-30), 7.01 (dd, 1H, *J* = 8.1, 1.2 Hz, H-12), 6.86 (s, 1H, H-14), 5.50 (s, 2H, H-24), 5.20 (dd, 2H, *J* = 66.5, 12.7 Hz, H-21), 2.84 (dt, 1H, *J* = 13.8, 6.9 Hz, H-15), 2.77–2.65 (m, 2H, H-7), 2.38 (s, 3H, H-31), 2.29 (d, 1H, *J* = 12.8 Hz, He-1), 2.19 (dd, 1H, *J* = 12.5, 1.8 Hz, H-5), 1.84–1.46 (m, 7H, Ha-1, H-2, H-3 and H-6), 1.26 (s, 3H, H-19), 1.25 (d, 6H, *J* = 6.9 Hz, H-16 and H-17), 1.23 (s, 3H, H-20); ^13^C NMR (150 MHz, CDCl_3_): δ 178.42, 146.76, 145.70, 143.47, 138.75, 134.64, 131.48, 129.81, 128.12, 126.88, 124.17, 123.92, 123.40, 57.76, 54.00, 47.56, 44.81, 37.90, 36.92, 36.37, 33.47, 29.93, 25.16, 24.00, 21.60, 21.19, 18.52, 16.47; ESI-MS *m/z*: 508.10 ([M + Na]^+^). Anal. Calcd for C_31_H_39_N_3_O_2_: C 76.67, H 8.09, N 8.65; found C 76.67, H 8.06, N 8.68.

*[1-(3-methoxybenzyl)-1H-1,2,3-triazole-4-yl]dehydroabietic acid methyl ester* (**3p**): Brown solid; yield 41.3%; m.p. 106.5–108.0 °C; ${\left[\alpha \right]}_{D}^{20}$+9.55 (c 0.012, CH_2_Cl_2_); IR (KBr, cm^−1^) 2929 (stretching, s, aliphatic C-H), 1712 (stretching, s, C=O); ^1^H NMR (600 MHz, CDCl_3_): δ 7.54 (s, 1H, H-23), 7.30 (t, 1H, *J* = 7.8 Hz and H-5), 7.16 (d, 1H, *J* = 8.2 Hz, H-11), 7.00 (d, 1H, *J =* 8.1, H-12), 6.91 (dd, 1H, *J* = 8.2, 2.2 Hz, H-28), 6.86 (m, 2H, H-14 and H-30), 6.81 (s, 1H, H-26), 5.51 (s, 2H, H-24), 5.21 (dd, 2H, *J* = 68.9, 12.7 Hz, H-21), 3.80 (s, 3H, H-31), 2.84 (dt, 1H, *J* = 13.8, 6.9 Hz, H-15), 2.75–2.71 (m, 2H, H-7), 2.29 (d, 1H, *J* = 12.5 Hz, He-1), 2.19 (dd, 1H, *J =* 12.4, 1.5 Hz, H-5), 1.90~1.46 (m, 7H, Ha-1, H-2, H-3 and H-6), 1.26 (s, 3H, H-19), 1.24 (d, 6H, *J =* 6.9 Hz, H-16 and H-17), 1.19 (s, 3H, H-20); ^13^C NMR (150 MHz, CDCl_3_): δ 178.57, 159.81, 146.41, 145.35, 143.22, 135.66, 134.30, 129.90, 126.57, 123.83, 123.59, 123.26, 119.88, 113.94, 113.32, 57.41, 54.96, 53.77, 47.22, 44.50, 37.58, 36.59, 36.04, 33.14, 29.58, 24.84, 23.68, 21.28, 18.19, 16.15; ESI-MS *m/z*: 523.30 ([M + Na]^+^). Anal. Calcd for C_31_H_39_N_3_O_3_: C 74.22, H 7.84, N 8.38; found C 74.20, H 7.85, N 8.40.

### 3.4. In Vitro Antiproliferative Evaluation

All the compounds (**3a**–**p**) were evaluated for their in vitro cytotoxicity against the following human cancer cell lines including CNE-2, HepG2, BEL-7402, Hela and normal liver cell HL-7702 by MTT assay. The cell lines were plated in 96-well plates at density of 5×10^3^ cells/well and maintained at 37 °C with 5% CO_2_. The target compounds **3a**–**p** dissolved in DMSO and further dilutions were made with DMEM, with cisplatin as the positive control. The concentration of the compounds used were 0.8, 4, 20, 100 μM. After the treatment of another 48 h and 72 h with different concentration of the samples (0.8, 4, 20, 100 μM), Therewith, 20 μL of MTT (5 mg/mL) was added and incubated for about 4 h. The medium was thrown away and the purple formazan precipitations were dissolved in 150 μL DMSO. The O. D. Value was measured at 490 nm in an enzyme labeling instrument [36,37].

## 4. Conclusions

Taken together, a series of dehydroabietic acid coupled 1,2,3-triazole derivatives (**3a**–**p**) were synthesized by a convenient one-pot Cu(I)-catalyzed Huisgen 1,3-dipolar cycloaddition reaction from propynyl ester **2** and a variety of readily available benzyl chloride/bromide without isolation of potentially unstable organic azide. The synthesized compounds were screened for cytotoxic activity against a panel of four human cancer cell lines and the human HL-7702 normal cell line using an MTT assay. Some compounds exhibited better anticancer activity against the tested cancer cell lines compared to positive controls cisplatin and low cytotoxicity on human normal liver cell HL-7702. Among these compounds, compounds **3c** (IC_50_ = 5.90 ± 0.41 µM) and **3k** (IC_50_ = 6.25 ± 0.37 μM) were the most promising derivatives. The antitumor activity in vivo and the mechanism in antitumor activity of dehydroabietic acid-1,2,3-triazole hybrids are under investigation.

## Figures and Tables

**Table 1:** Antiproliferative activity of dehydroabietic acid-based 1,2,3-triazole compounds ^a^.

Compound	IC_50_ (μM)				
	CNE-2	HepG2	BEL-7402	HeLa	HL-7702
**3a** (R = H)	33.40 ± 0.33	>100	36.44 ± 070	67.88 ± 0.56	>100
**3b** (R = *o*-F)	21.44 ± 0.52	25.86 ± 0.27	18.63 ± 0.82	17.76 ± 0.31	>100
**3c** (R = *m*-F)	10.92 ± 0.21	5.90 ± 0.41	16.72 ± 0.06	20.05 ± 0.54	>100
**3d** (R = *p*-F)	12.20 ± 0.33	20.60 ± 0.09	14.84 ± 075	22.33 ± 0.35	>100
**3e** (R = *o*-Cl)	74.88 ± 0.14	45.08 ± 0.33	25.06 ± 0.32	44.28 ± 0.43	>100
**3f** (R = *m*-Cl)	59.03 ± 0.28	22.27 ± 0.35	26.09 ± 0.15	53.78 ± 0.32	>100
**3g** (R = *p*-Cl)	60.73 ± 066	>100	38.04 ± 0.48	>100	>100
**3h** (R = *o*-Br)	48.30 ± 0.27	35.42 ± 0.21	27.26 ± 0.36	25.90 ± 0.20	>100
**3i** (R = *m*-Br)	44.14 ± 0.22	40.66 ± 0.62	36.88 ± 0.23	56.08 ± 0.36	>100
**3j** (R = *p*-Br)	>100	23.40 ± 0.32	14.53 ± 0.62	43.18 ± 0.22	>100
**3k** (R = *o*-NO_2_)	44.90 ± 0.32	6.25 ± 0.37	18.62 ± 0.26	>100	>100
**3l** (R = *m*-NO_2_)	11.45 ± 0.18	15.83 ± 0.64	15.39 ± 0.51	67.37 ± 0.33	>100
**3m** (R = *p*-NO_2_)	19.61 ± 0.38	>100	22.81 ± 0.22	22.48 ± 0.35	>100
**3n** (R = *m*-CH_3_)	>100	60.18 ± 0.39	23.61 ± 0.44	25.32 ± 0.81	>100
**3o** (R = *p*-CH_3_)	>100	51.78 ± 0.43	41.89 ± 0.72	42.51 ± 0.37	>100
**3p** (R = *m*-OCH_3)_	80.98 ± 0.78	>100	25.03 ± 0.22	24.66 ± 0.16	>100
DHAA	88.64 ± 0.73	80.36 ± 0.84	46.70 ± 0.55	37.40 ± 0.64	>100
Cisplatin	8.75 ± 0.24	6.42 ± 0.18	12.68 ± 0.33	1.94 ± 0.20	20.76 ± 0.83

^a^ IC_50_ values are expressed as the mean ± SD (standard deviation) from three independent experiments. The results indicate statistically significant differences at *p* < 0.05.

## Supplementary Materials

The Supplementary Materials are available online.

## References

[1] Chen H., Lei H.M., Ma B., Liu M., Guo D., Liu X., Hu L.H. (2016). Synthesis and cytotoxicity evaluation of 4′-amino-4′-dehydroxyloleandrin derivatives. Fitoterapia.

[2] Gao F., Zhang X., Wang T.F., Xiao J.Q. (2019). Quinolone hybrids and their anti-cancer activities: An overview. Eur. J. Med. Chem..

[3] Zhuang C.L., Guan X.H., Ma H., Cong H., Zhang W.N., Miao Z.Y. (2019). Small molecule-drug conjugates: A novel strategy for cancer-targeted treatment. Eur. J. Med. Chem..

[4] Gao J., Chen M., Ren X.C., Zhou X.B., Shang Q., Lu W.Q., Luo P., Jiang Z.H. (2017). Synthesis and cardiomyocyte protection activity of crocetin diamide derivatives. Fitoterapia.

[5] González M.A. (2015). Aromatic abietane diterpenoids: Their biological activity and synthesis. Nat. Prod. Rep..

[6] González M.A. (2015). Aromatic abietane diterpenoids: Total syntheses and synthetic studies. Tetrahedron.

[7] González M.A. (2014). Synthetic derivatives of aromatic abietane diterpenoids and their biological activities. Eur. J. Med. Chem..

[8] Fonseca T., Gigante B., Marques M.M. (2004). Synthesis and antiviral evaluation of benzimidazoles, quinoxalines and indoles from dehydroabietic acid. Bioorg. Med. Chem..

[9] Kang M.S., Hirai S., Goto T. (2008). Dehydroabietic acid, a phytochemical, acts as ligand for PPARs inmacrophages and adipocytes to regulate inflammation. Biochem. Biophys. Res. Commun..

[10] Helfenstein A., Vahermo M., Nawrot D.A., Demirci F., Iscan G., Krogerus S., Yli-Kauhaluoma J., Moreira V.M., Tammela P. (2016). Antibacterial profiling of abietane-type diterpenoids. Bioorg. Med. Chem..

[11] Zhang W.M., Yang T., Pan X.Y., Liu X.L., Lin H.X., Gao Z.B., Yang C.G., Cui Y.M. (2017). The synthesis and antistaphylococcal activity of dehydroabietic acid derivatives: Modifications at C12 and C7. Eur. J. Med. Chem..

[12] Liu M.L., Pan X.Y., Yang T., Zhang W.M., Wang T.Q., Wang H.Y., Lin H.X., Yang C.G., Cui Y.M. (2016). The synthesis and antistaphylococcal activity of dehydroabietic acid derivatives: Modifications at C-12. Bioorg. Med. Chem. Lett..

[13] Berger M., Roller A., Maulide N. (2017). Synthesis and antimicrobial evaluation of novel analogues of dehydroabietic acid prepared by C-H-Activation. Eur. J. Med. Chem..

[14] Chen N.Y., Duan W.G., Lin G.S., Liu L.Z., Zhang R., Li D.P. (2016). Synthesis and antifungal activity of dehydroabietic acid-based 1,3,4-thiadiazole-thiazolidinone compounds. Mol. Divers..

[15] Pertino M.W., Vega C., Rolón M., Coronel C., Arias A.R., Hirschmann G.S. (2017). Antiprotozoal activity of triazole derivatives of dehydroabietic acid and oleanolic acid. Molecules.

[16] González M.A., Guaita D.P., Royero J.C., Zapata B., Agudelo L., Arango A.M., Galvis L.B. (2010). Synthesis and biological evaluation of dehydroabietic acid derivatives. Eur. J. Med. Chem..

[17] Wada H., Kodato S., Kawamori M., Morikawa T., Nakai H., Takeda M., Saito S., Onoda Y., Tamaki H. (1985). Antiulcer activity of dehydroabietic acid derivatives. Chem. Pharm. Bull..

[18] Roa-Linares V.C., Brand Y.M., Agudelo-Gomez L.S., Tangarife-Castaño V., Betancur-Galvis L.A., Gallego-Gomez J.C., González M.A. (2016). Anti-herpetic and anti-dengue activity of abietane ferruginol analogues synthesized from (+)-dehydroabietylamine. Eur. J. Med. Chem..

[19] Kim J., Kang Y.G., Lee J.Y., Choi D.H., Cho Y.U., Shin J.M., Park J.S., Lee J.H., Kim W.G., Seo D.B. (2015). The natural phytochemical dehydroabietic acid is an anti-aging reagent that mediates the direct activation of SIRT1. Mol. Cell. Endocrinol..

[20] Huang X.C., Huang R.Z., Liao Z.X., Pan Y.M., Gou S.H., Wang H.S. (2016). Synthesis and pharmacological evaluation of dehydroabietic acid thiourea derivatives containing bisphosphonate moiety as an inducer of apoptosis. Eur. J. Med. Chem..

[21] Luo D.J., Ni Q., Ji A.L., Gu W., Wu J.H., Jiang C.P. (2016). Dehydroabietic acid derivative QC4 induces gastric cancer cell death via oncosis and apoptosis. BioMed Res. Int..

[22] Huang X.C., Wang M., Wang H.S., Chen Z.F., Zhang Y., Pan Y.M. (2014). Synthesis and antitumor activities of novel dipeptide derivatives derived from dehydroabietic acid. Bioorg. Med. Chem. Lett..

[23] Reddy T.S., Kulhari H., Reddy V.G., Subba-Rao A.V., Bansal V., Kamal A., Shukla R. (2015). Synthesis and biological evaluation of pyrazolo–triazole hybrids as cytotoxic and apoptosis inducing agents. Org. Biomol. Chem..

[24] Stefely J.A., Palchaudhuri R., Miller P.A., Peterson R.J., Moraski G.C., Hergenrother P.J., Miller M.J. (2010). *N*-((1-Benzyl-1*H*-1,2,3-triazol-4-yl)methyl)arylamide as a new scaffold that provides rapid access to antimicrotubule agents: Synthesis and evaluation of antiproliferative activity against select cancer cell Lines. J. Med. Chem..

[25] Binh L.H., Van N.T.T., Kien V.T., My N.T.T., van Chinh L., Nga N.T., Tien H.X., Thao D.T., Vu T.K. (2016). Synthesis and in vitro cytotoxic evaluation of new triazole derivatives based on artemisinin via click chemistry. Med. Chem. Res..

[26] Valdomir G., de los Ángeles Fernández M., Lagunes I., Padron J.I., Martin V.S., Padron J.M., Davyt D. (2018). Oxa/thiazole-tetrahydropyran triazole-linked hybrids with selective antiproliferative activity against human tumour cells. New J. Chem..

[27] Reddy V.G., Reddy T.S., Nayak V.L., Prasad B., Reddy A.P., Ravikumar A., Taj S., Kamal A. (2016). Design, synthesis and biological evaluation of *N*-((1-benzyl-1*H*-1,2,3-triazol-4-yl)methyl)-1,3-diphenyl-1*H*-pyrazole-4-carboxamides as CDK1/Cdc2 inhibitors. Eur. J. Med. Chem..

[28] Yadav P., Lal K., Kumar A., Guru S.K., Jaglan S., Bhushan S. (2017). Green synthesis and anticancer potential of chalcone linked-1,2,3-triazoles. Eur. J. Med. Chem..

[29] Tron G.C., Pirali T., Billington R.A., Canonico P.L., Sorba G., Genazzani A.A. (2008). Click chemistry reactions in medicinal chemistry: Applications of the 1,3-dipolar cycloaddition between azides and alkynes. Med. Res. Rev..

[30] Agalave S.G., Maujan S.R., Pore V.S. (2011). Click Chemistry: 1,2,3-triazoles as pharmacophores. Chem.-Asian J..

[31] Wang X., Pang F.H., Huang L., Yang X.P., Ma X.L., Jiang C.N., Li F.Y., Lei F.H. (2018). Synthesis and Biological Evaluation of Novel Dehydroabietic Acid-Oxazolidinone Hybrids for Antitumor Properties. Int. J. Mol. Sci..

[32] Chen N.Y., Duan W.G., Liu L.Z., Li F.Y., Lu M.P., Liu B.M. (2015). Synthesis and antifungal activity of dehydroabietic acid-based thiadiazole-phosphonates. Holzforschung.

[33] Li F.Y., Wang X., Duan W.G., Lin G.S. (2017). Synthesis and in vitro anticancer activity of novel dehydroabietic acid-based acylhydrazones. Molecules.

[34] Lin G.S., Duan W.G., Yang L.X., Huang M., Lei F.H. (2017). Synthesis and antifungal activity of novel myrtenal-based 4-methyl-1,2,4-triazole-thioethers. Molecules.

[35] Gu W., Miao T.T., Hua D.W., Jin X.Y., Tao X.B., Huang C.B., Wang S.F. (2017). Synthesis and in vitro cytotoxic evaluation of new 1H-benzo[d]imidazole derivatives of dehydroabietic acid. Bioorg. Med. Chem. Lett..

[36] Huang R.Z., Liang G.B., Huang X.C., Zhang B., Zhou M.M., Liao Z.X., Wang H.S. (2017). Discovery of dehydroabietic acid sulfonamide based derivatives as selective matrix metalloproteinases inactivators that inhibit cell migration and proliferation. Eur. J. Med. Chem..

[37] Nagarsenkar A., Guntuku L., Guggilapu S.D., Bai K.D., Srinivasulu G., Naidu V.G.M., Babu B.N. (2016). Synthesis and apoptosis inducing studies of triazole linked 3-benzylidene isatin derivatives. Eur. J. Med. Chem..

