# Supplementary material for: Synthesis and Antiproliferative Evaluation of Novel Hybrids of Dehydroabietic Acid Bearing 1,2,3-Triazole Moiety
Source: Molecules. 2019 Nov 19;24(22):4191. doi: 10.3390/molecules24224191 (PMC6891475; doi:10.3390/molecules24224191)
Supplement: Supplementary file 1 [file molecules-24-04191-s001.pdf]

Synthesis and antiproliferative evaluation of novel hybrids of dehydroabietic acid bearing 1,2,3-triazole moiety

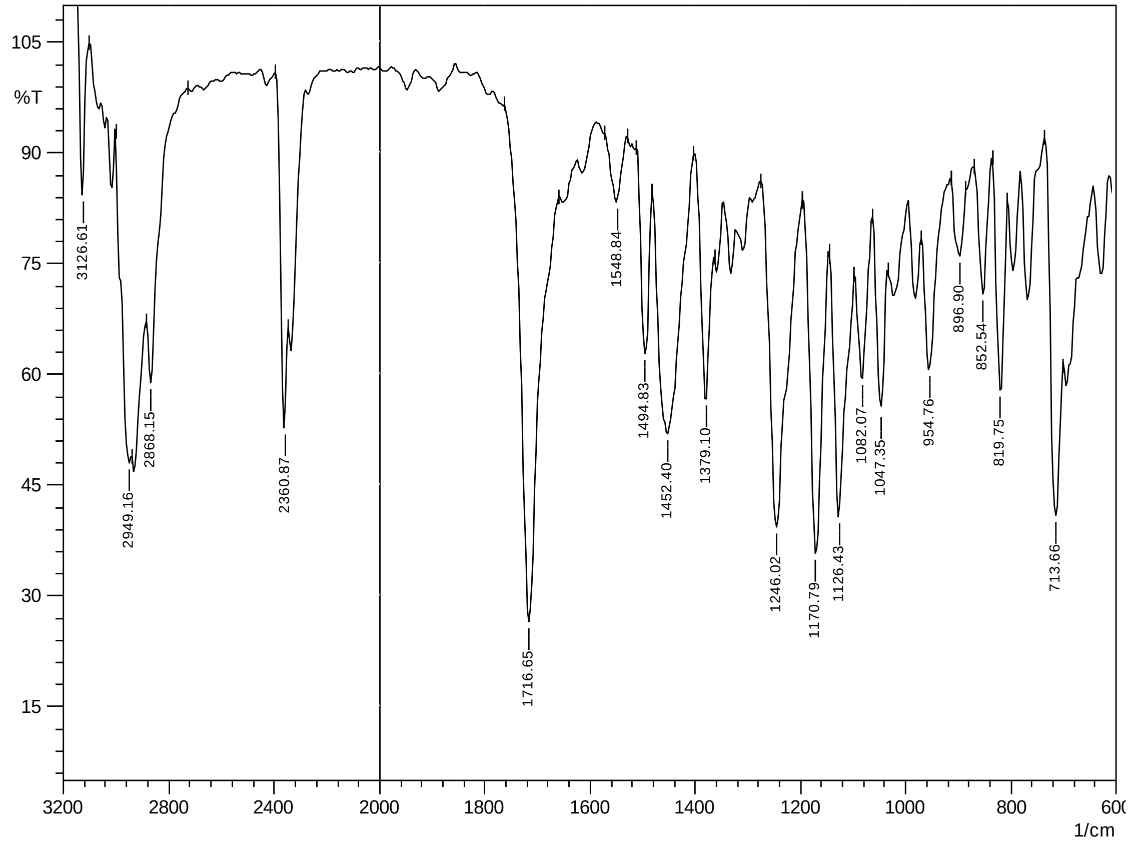

Figure S1. FTIR spectrum of **3a**

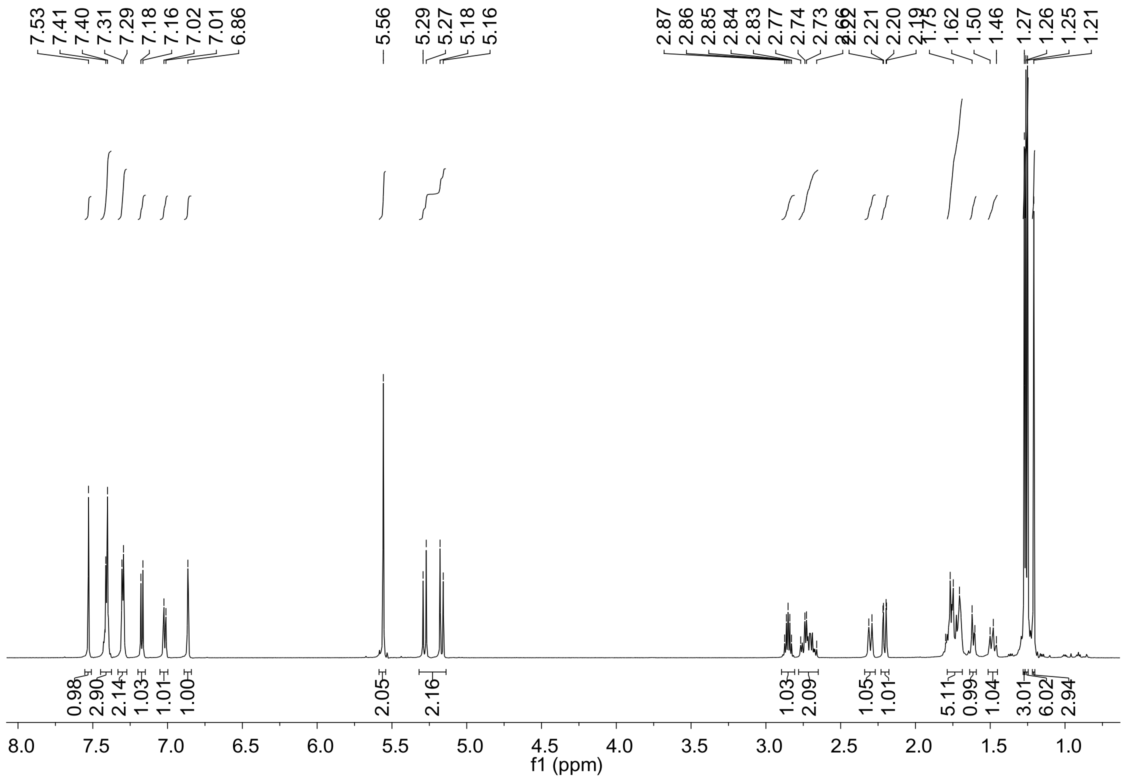

Figure S2. ^{1}H-NMR spectrum of **3a**

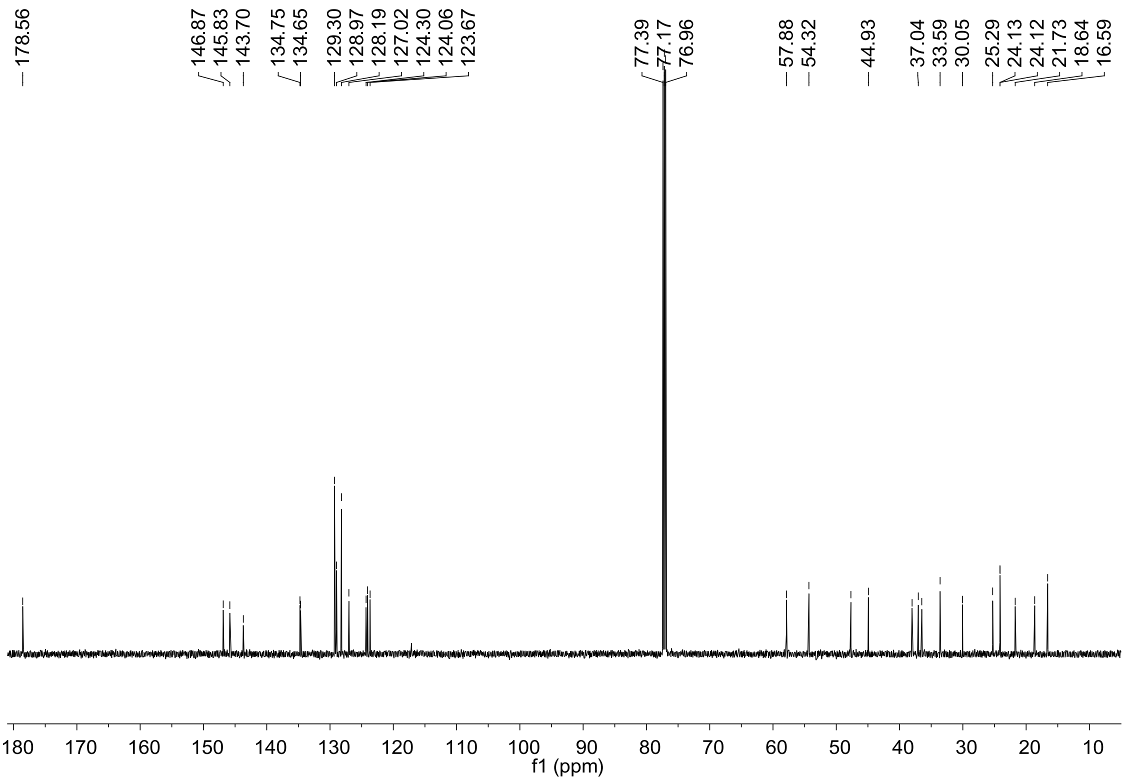

Figure S3. ^{13}C-NMR spectrum of **3a**

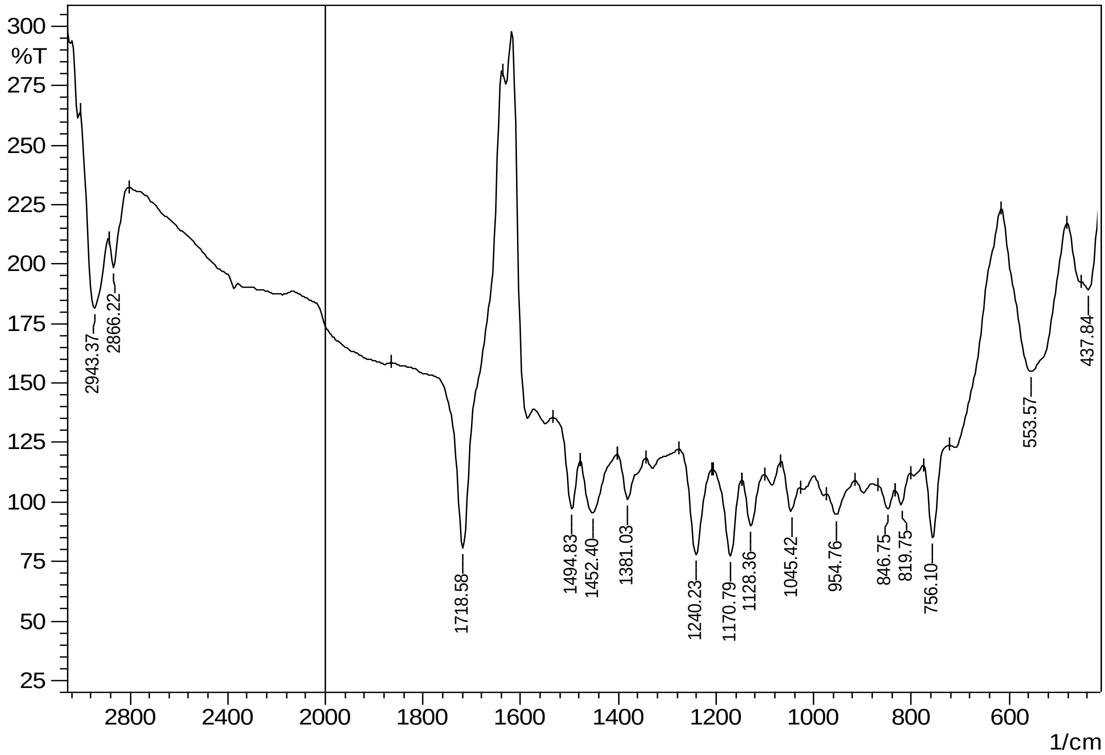

Figure S4. FTIR spectrum of **3b**

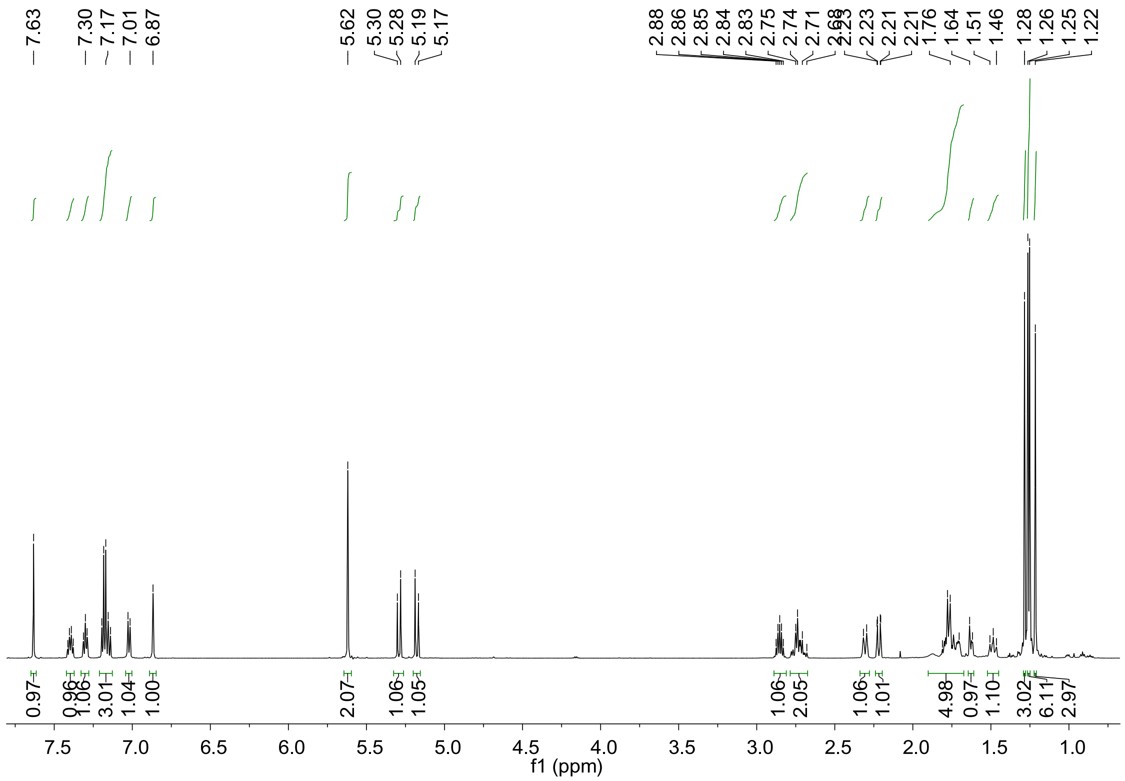

Figure S5. ^{1}H-NMR spectrum of **3b**

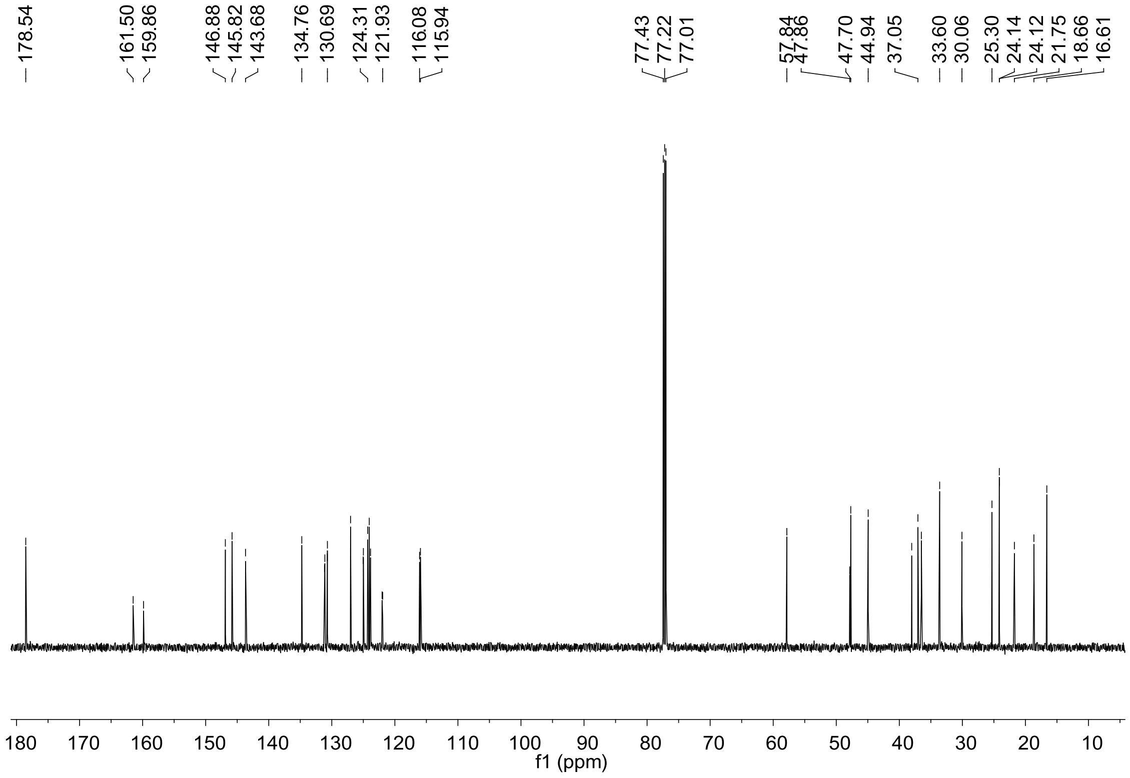

Figure S6. ^{13}C-NMR spectrum of **3b**

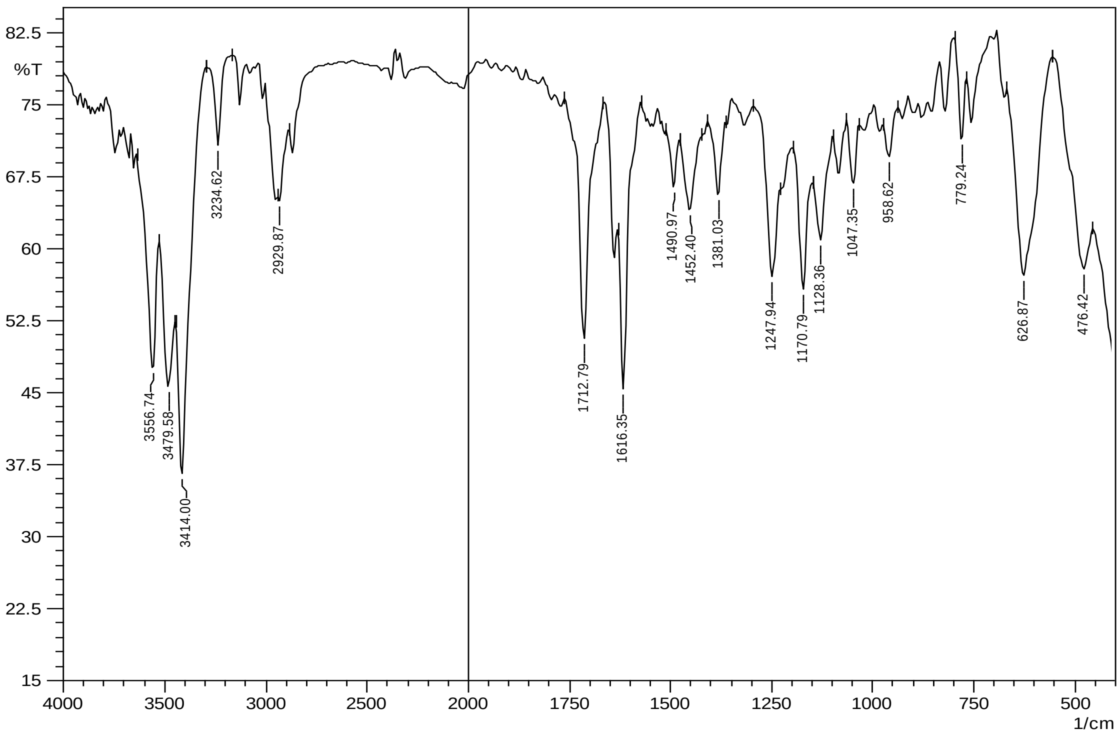

Figure S7. FTIR spectrum of **3c**

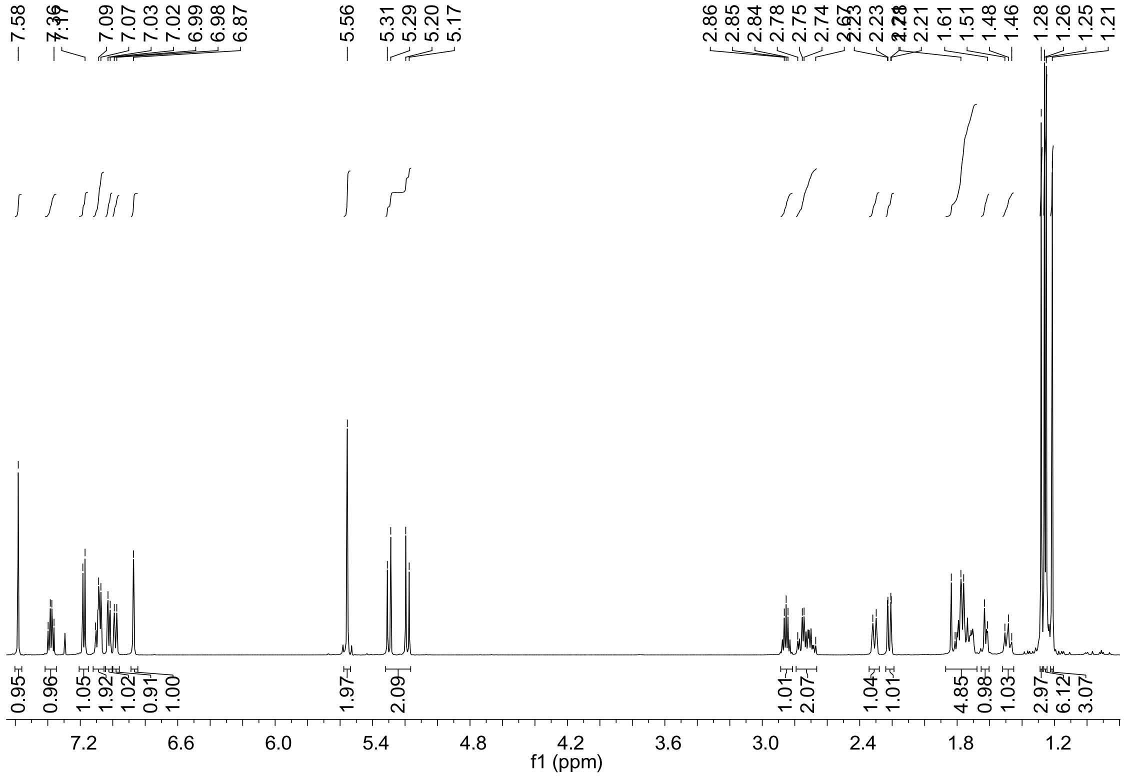

Figure S8. ^{1}H-NMR spectrum of **3c**

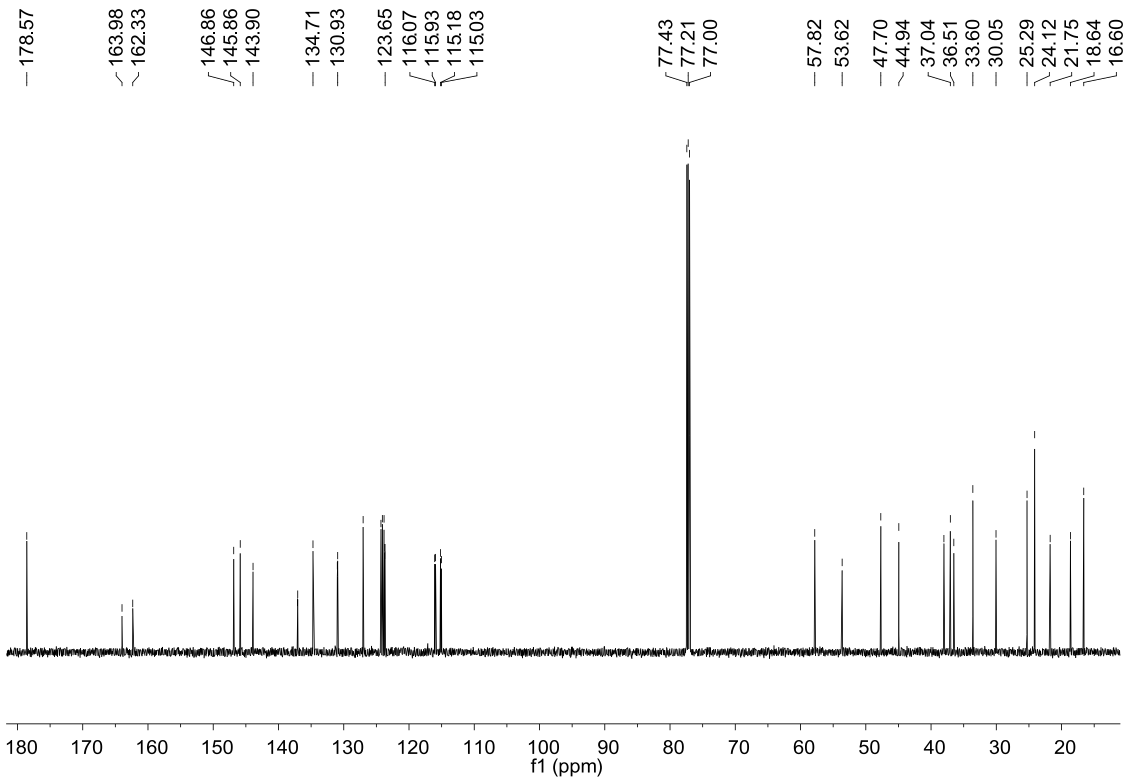

Figure S9. ^{13}C-NMR spectrum of **3c**

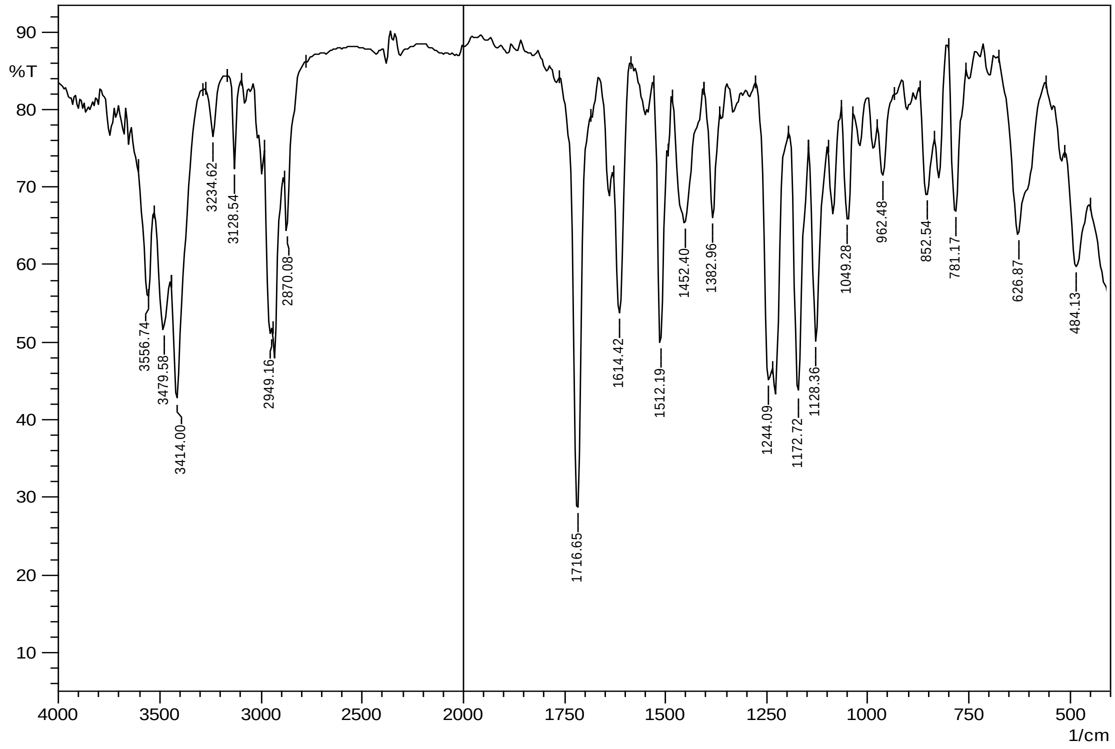

Figure S10. FTIR spectrum of **3d**

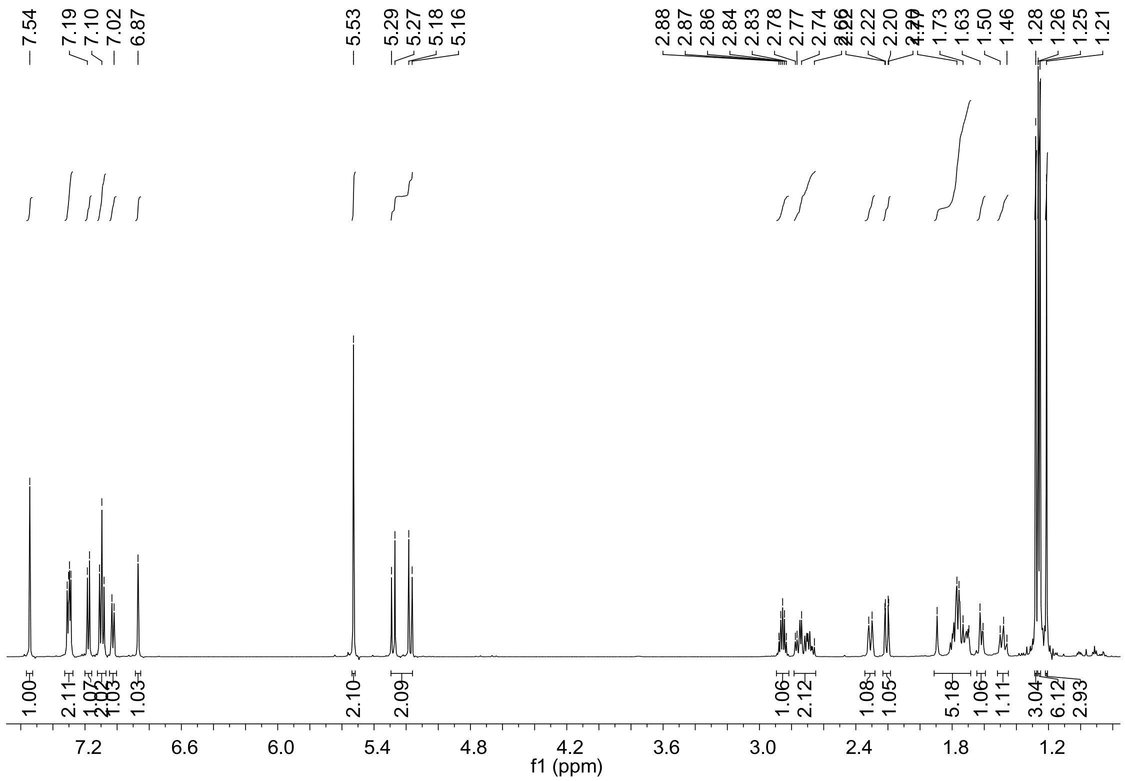

Figure S11. ^{1}H-NMR spectrum of **3d**

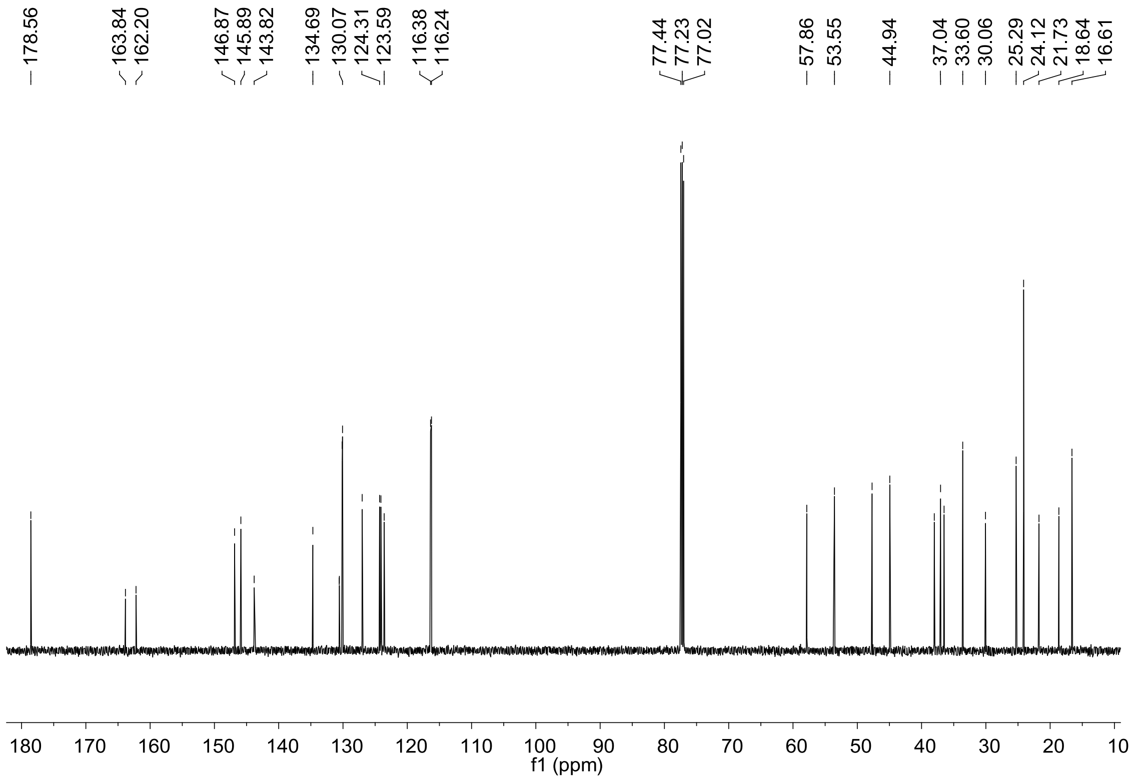

Figure S12. ^{13}C-NMR spectrum of **3d**

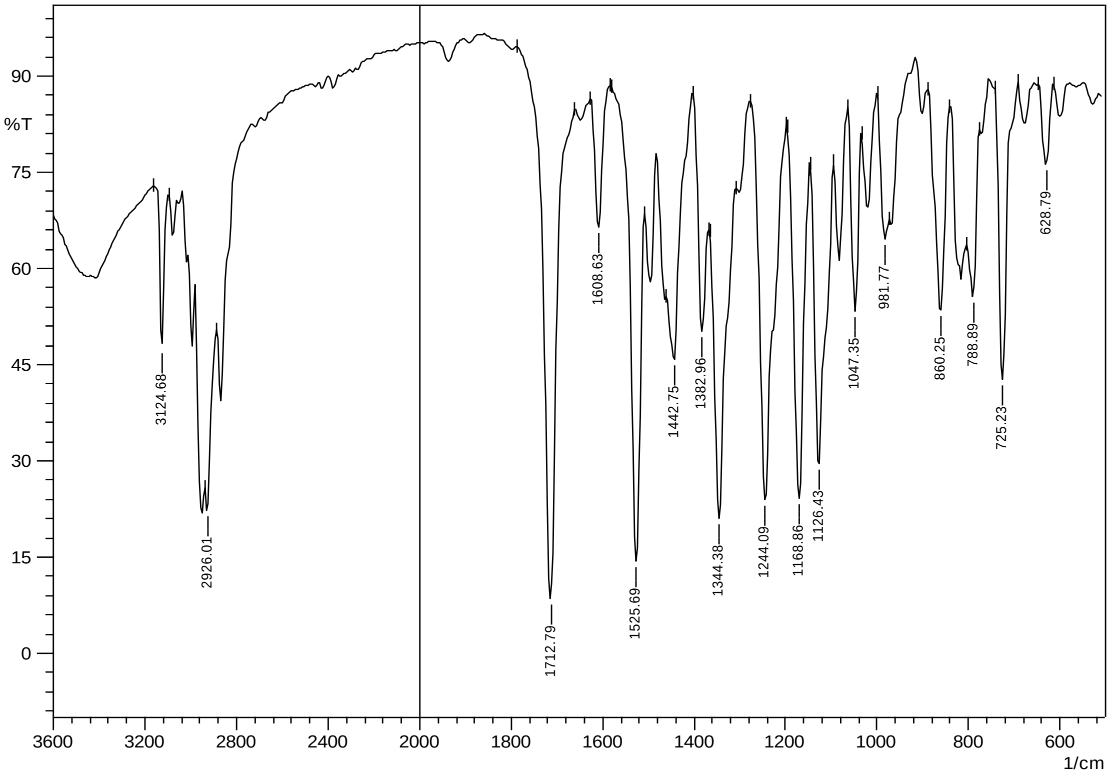

Figure S13. FTIR spectrum of **3e**

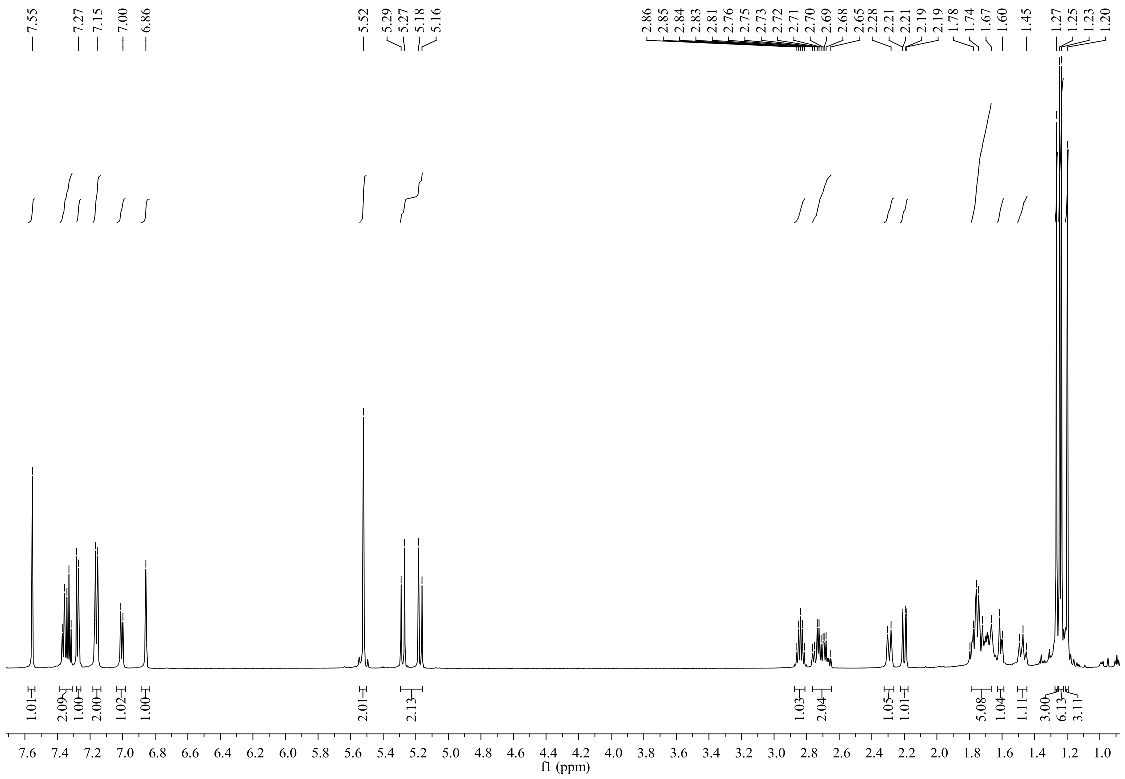

Figure S14. ^{1}H-NMR spectrum of **3e**

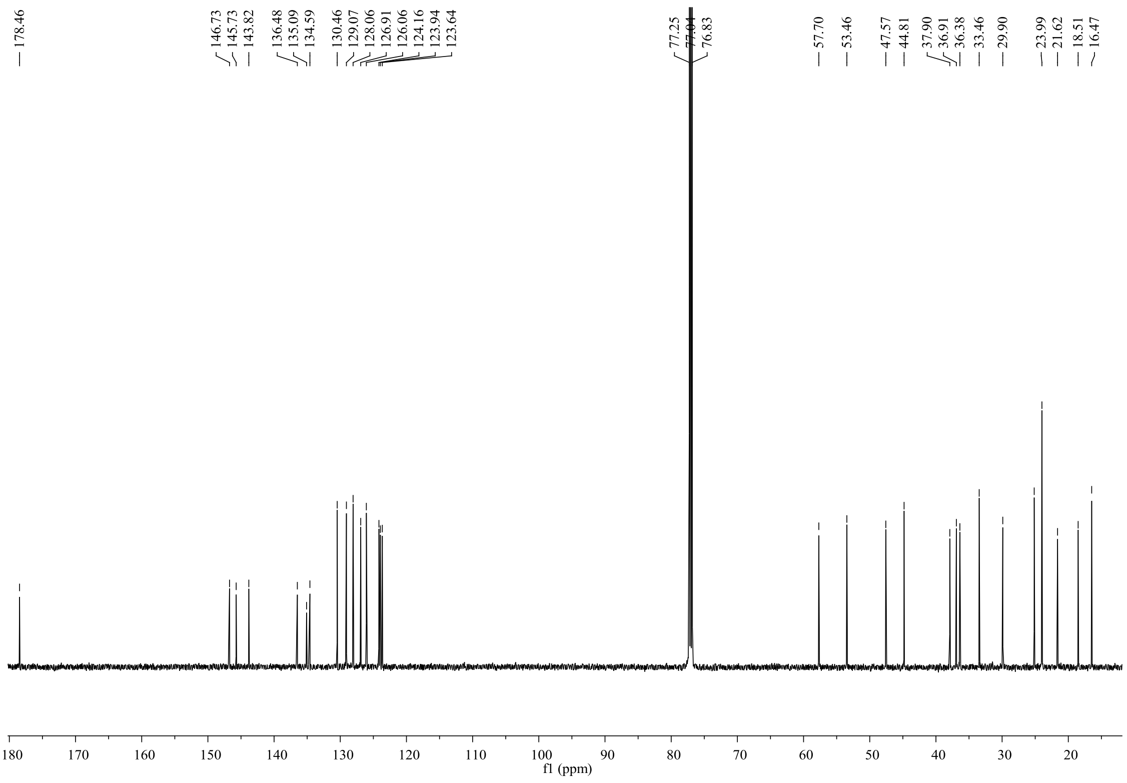

Figure S15. ^{13}C-NMR spectrum of **3e**

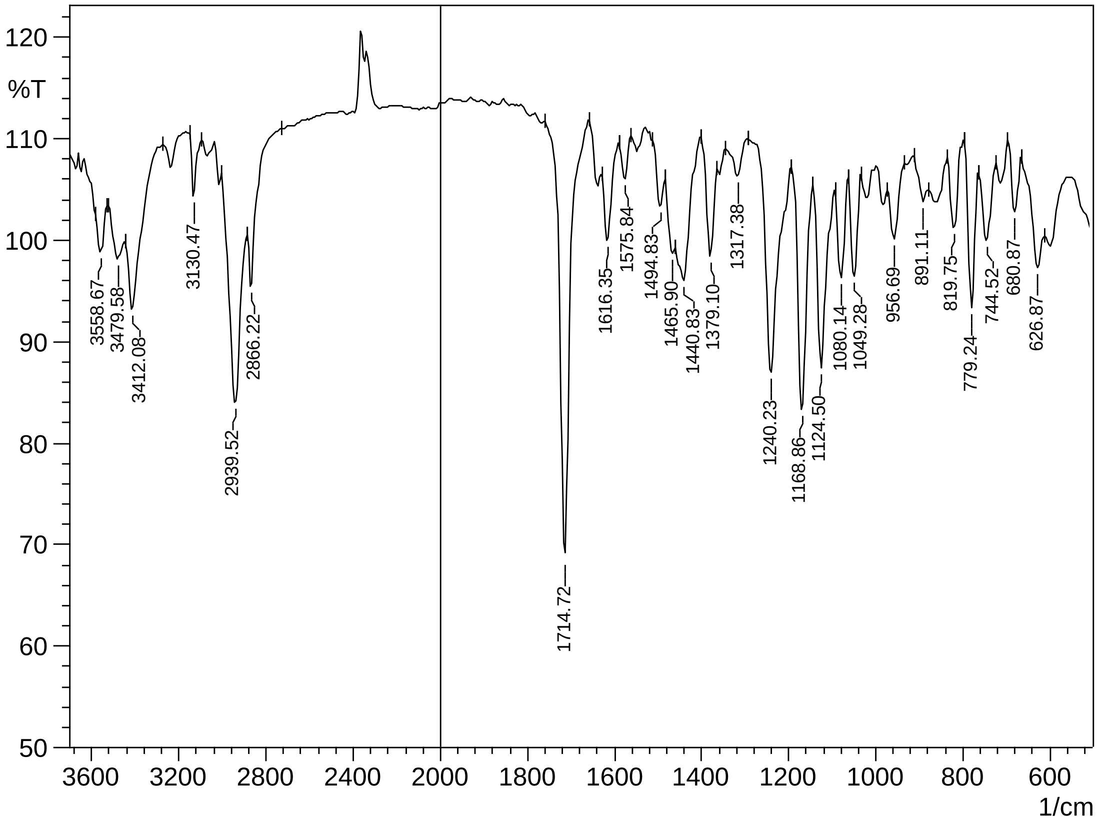

Figure S16. FTIR spectrum of **3f**

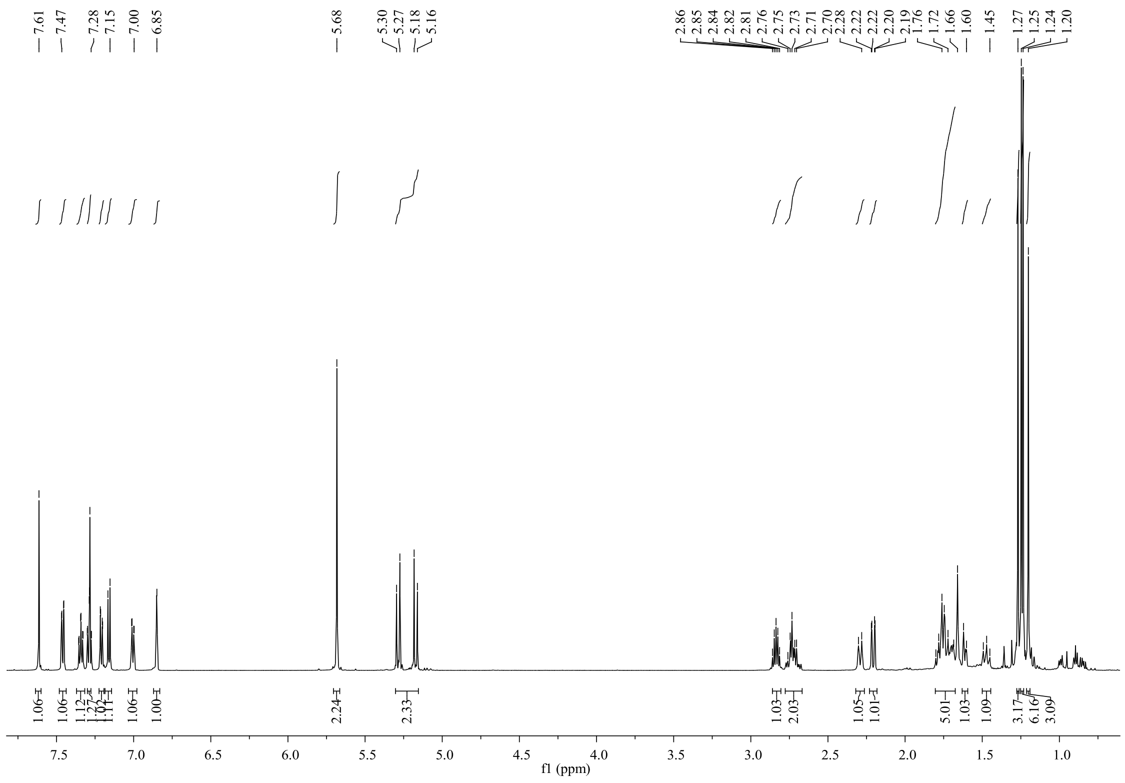

Figure S17. ^{1}H-NMR spectrum of **3f**

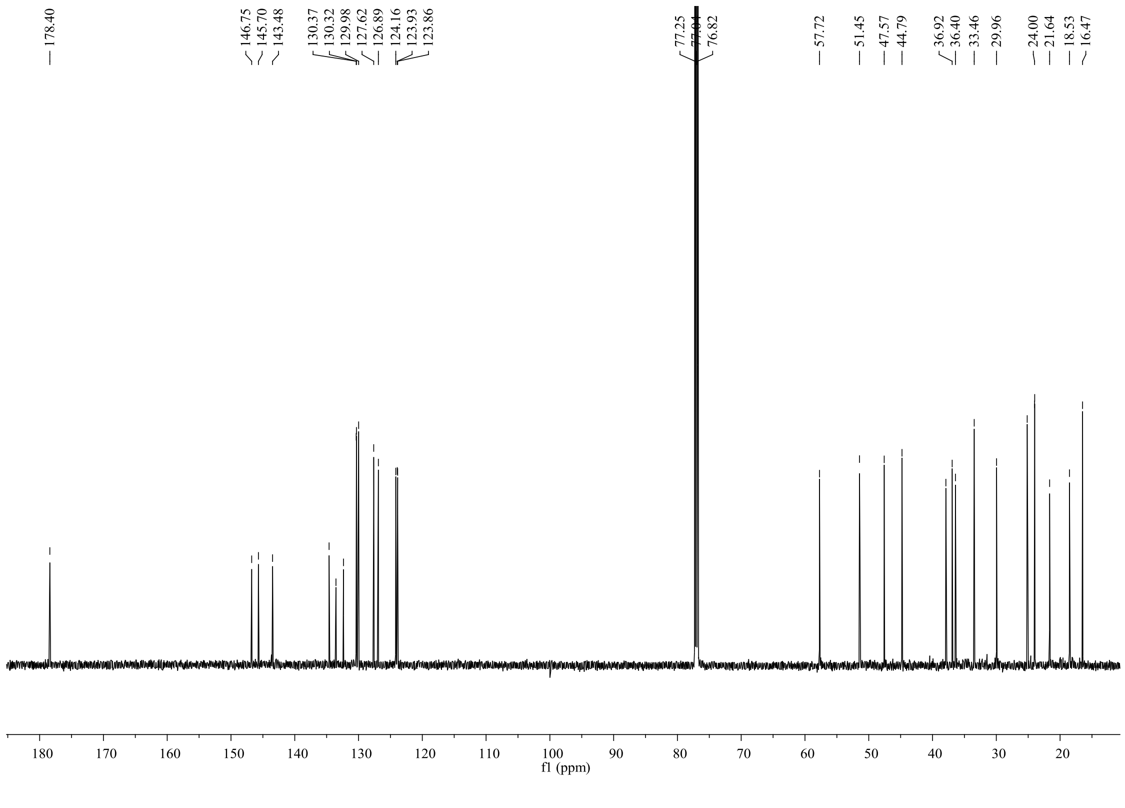

Figure S18. ^{13}C-NMR spectrum of **3f**

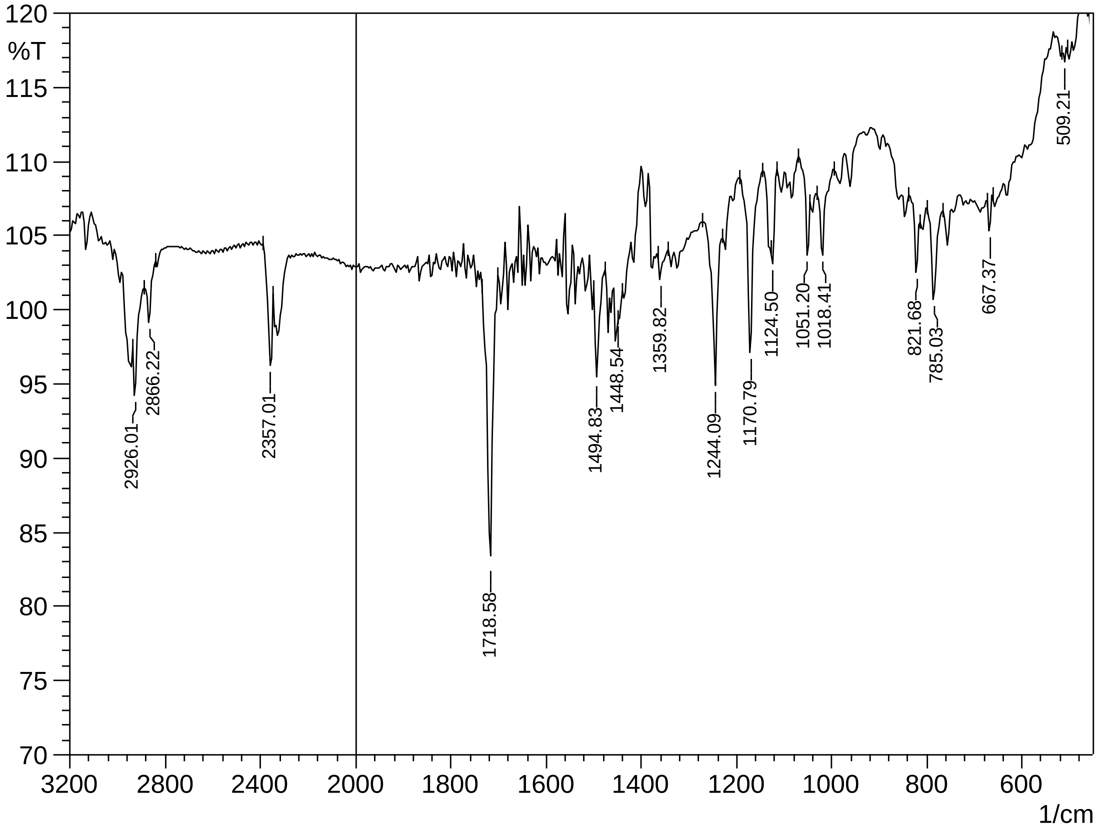

Figure S19. FTIR spectrum of **3g**

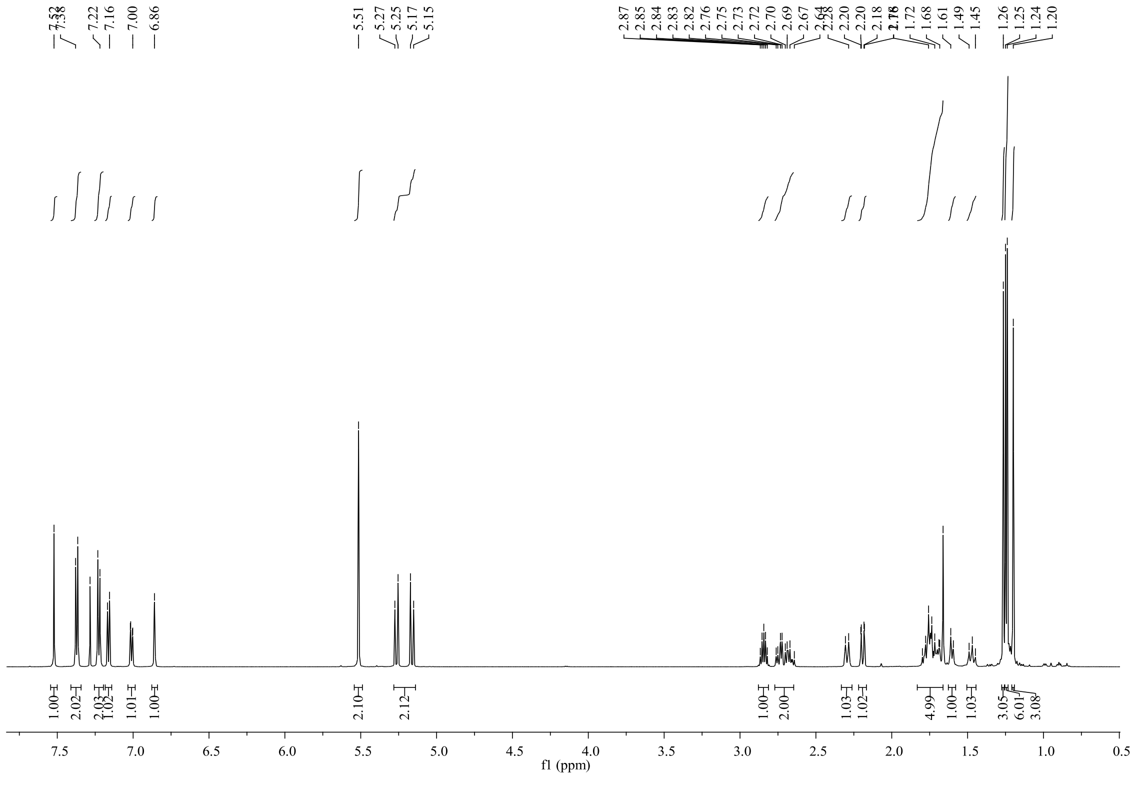

Figure S20. ^{1}H-NMR spectrum of **3g**

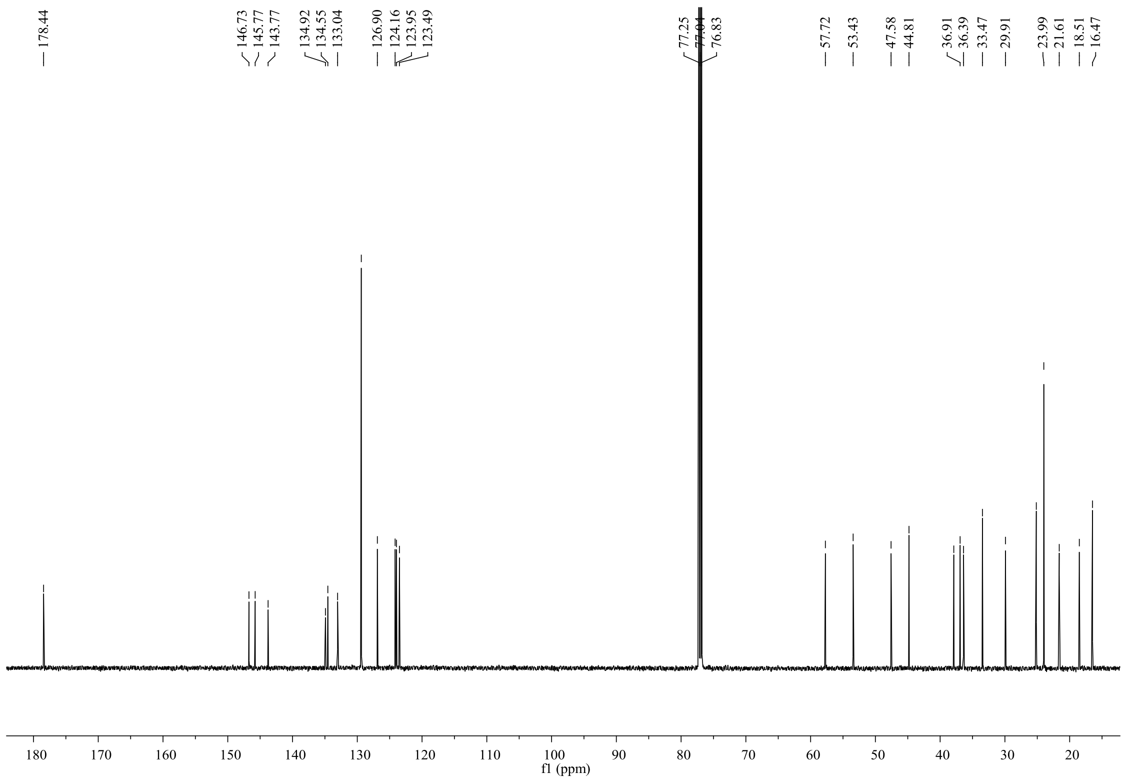

Figure S21. ^{13}C-NMR spectrum of **3g**

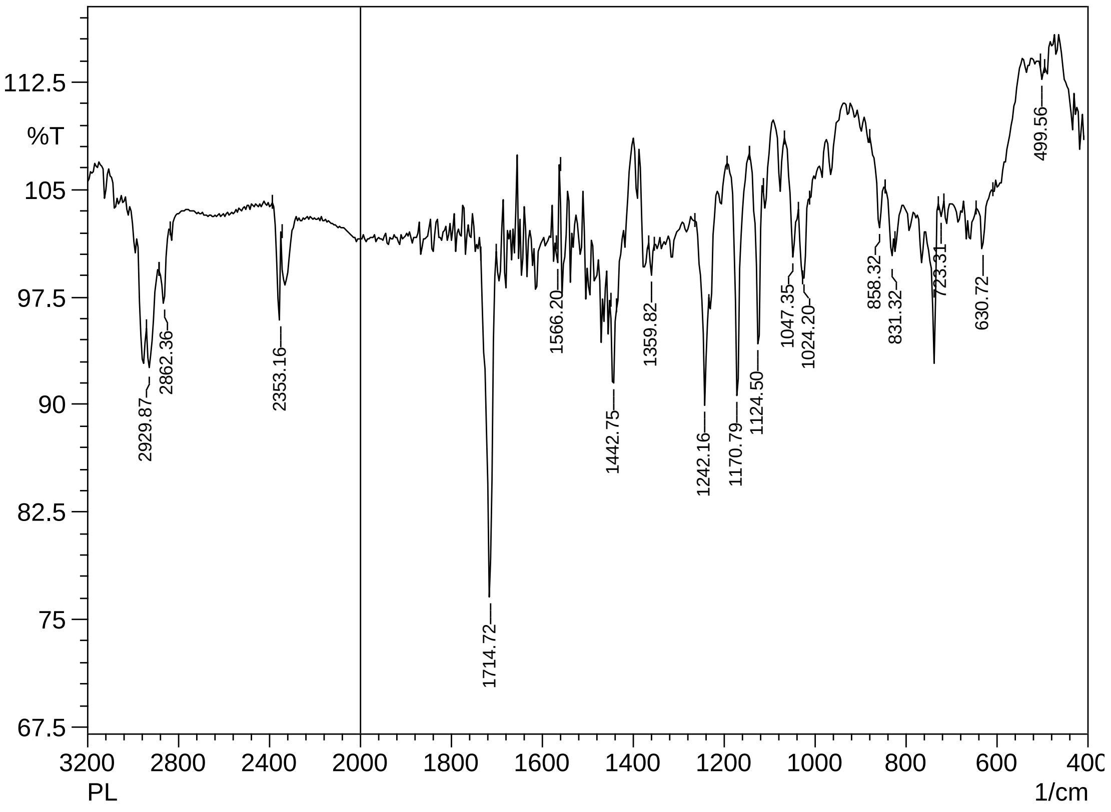

Figure S22. FTIR spectrum of **3h**

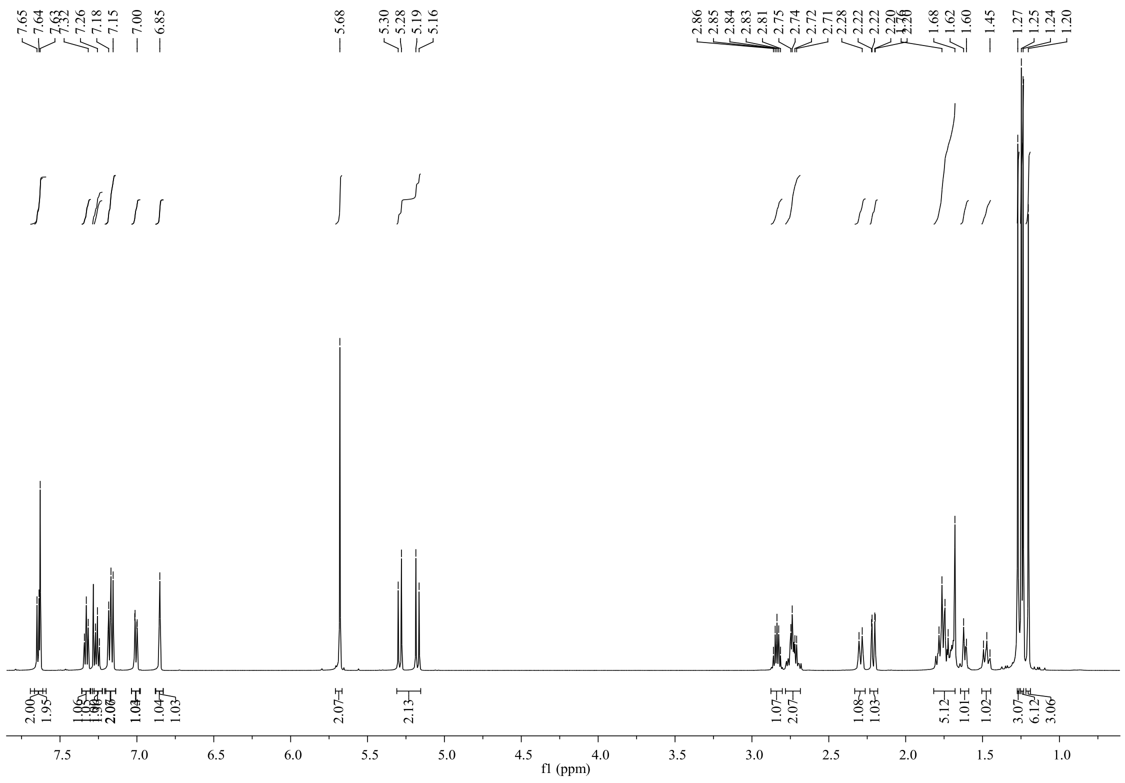

Figure S23. ^{1}H-NMR spectrum of **3h**

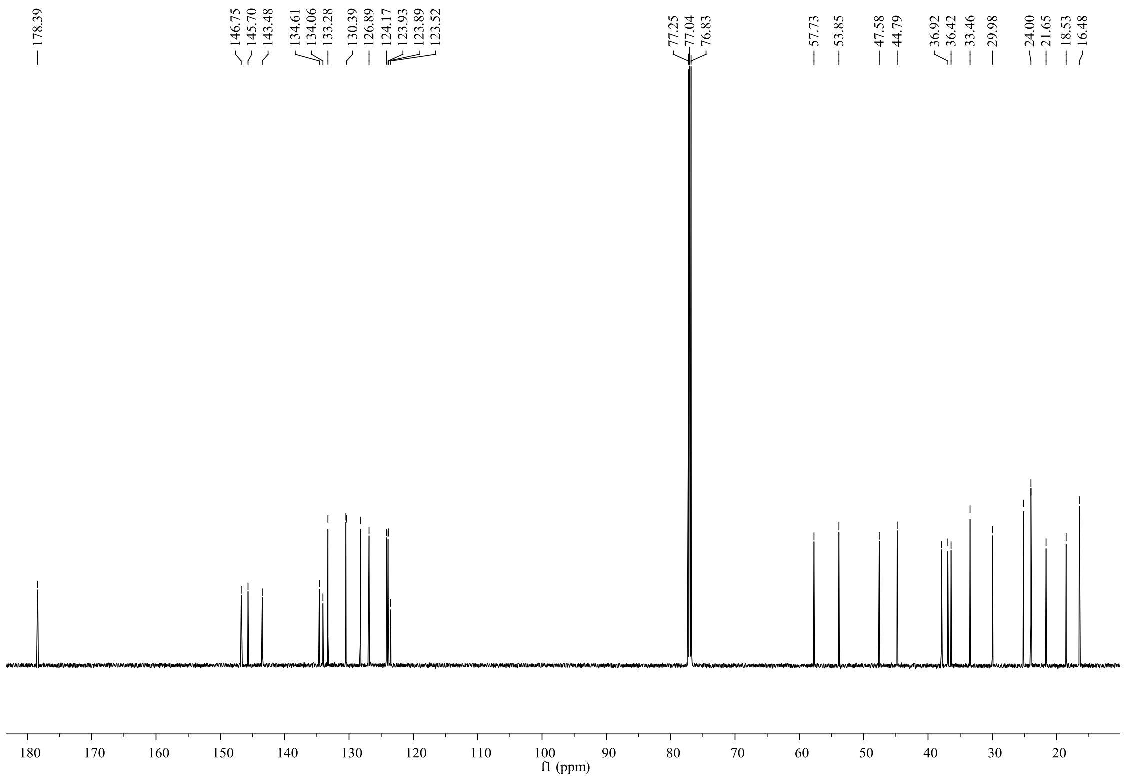

Figure S24. ^{13}C-NMR spectrum of **3h**

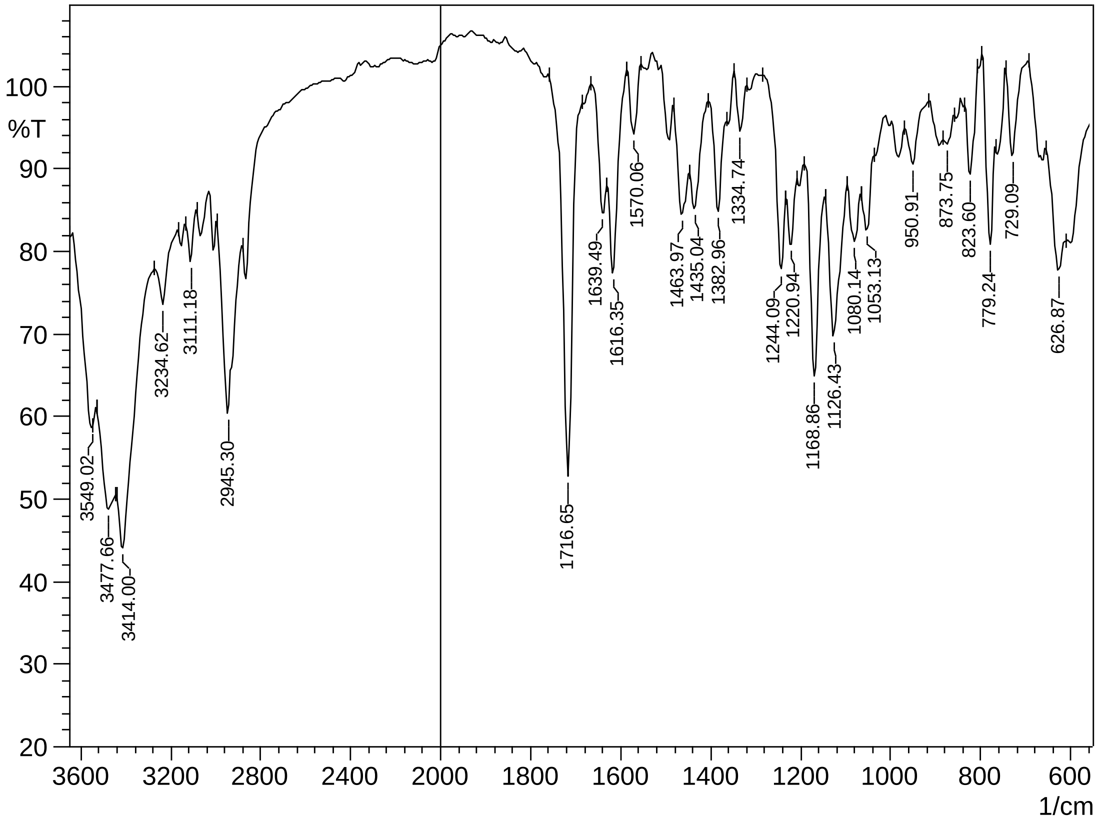

Figure S25. FTIR spectrum of **3i**

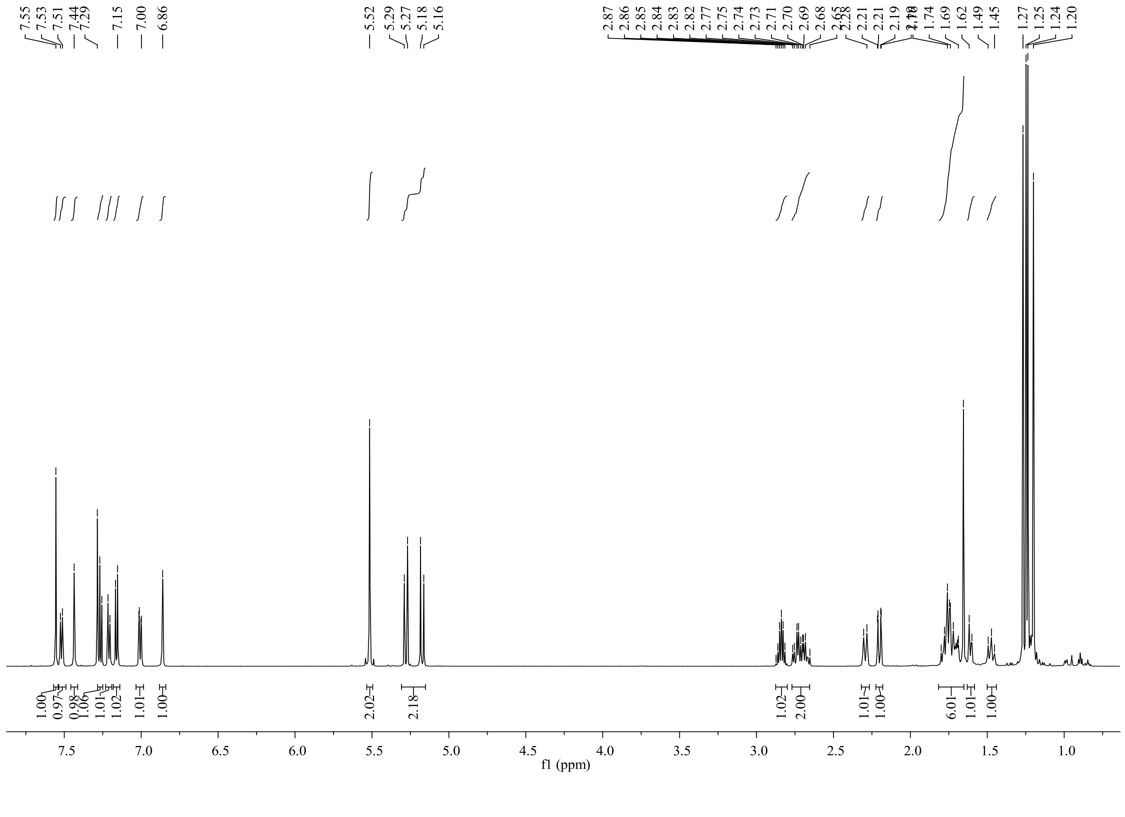

Figure S26. ^{1}H-NMR spectrum of **3i**

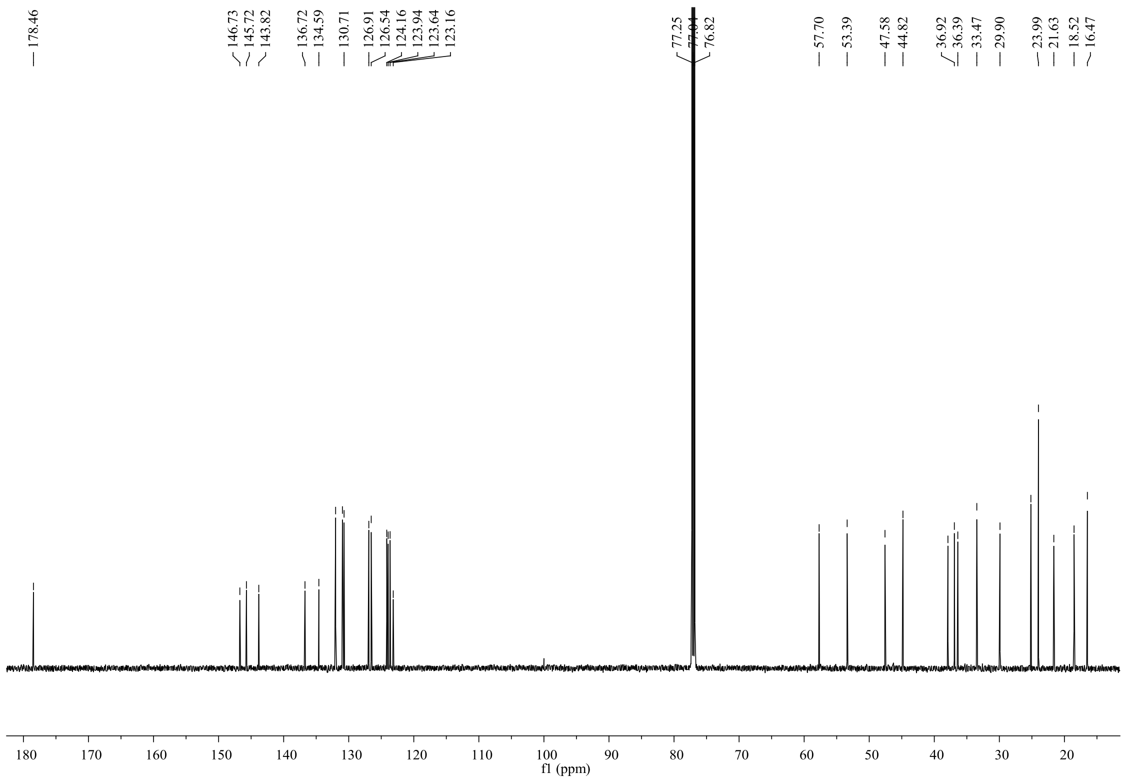

Figure S27. ^{13}C-NMR spectrum of **3i**

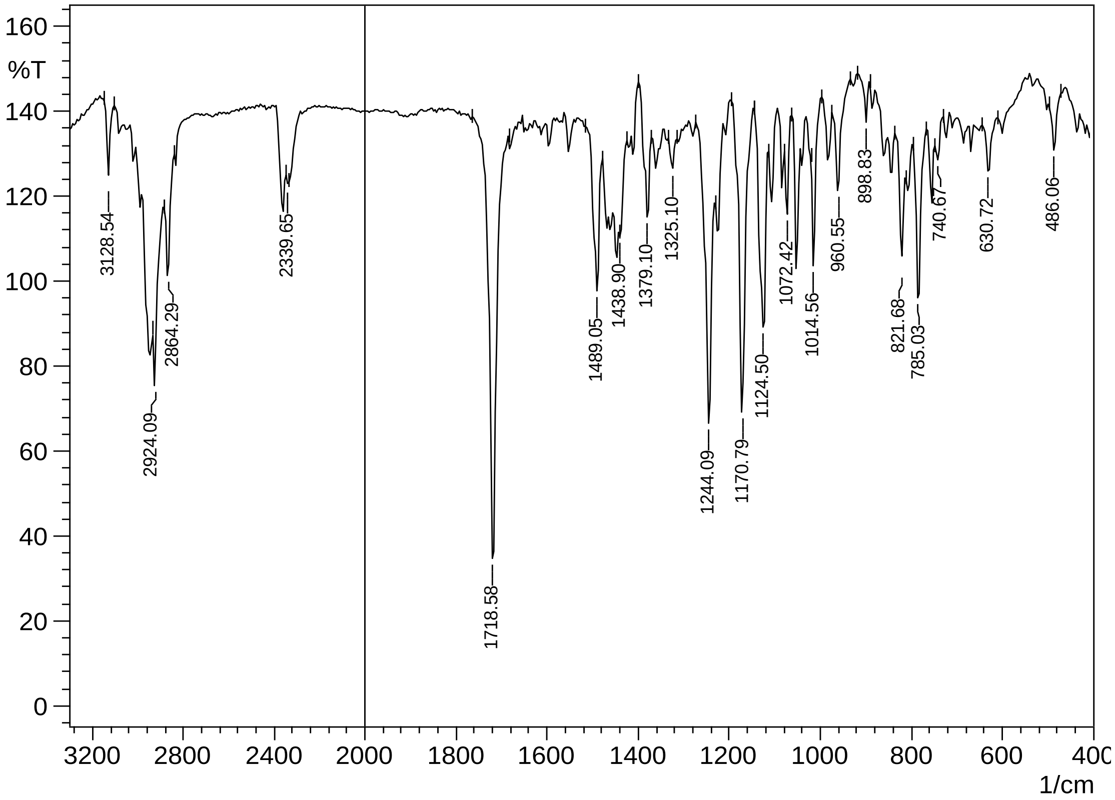

Figure S28. FTIR spectrum of **3j**

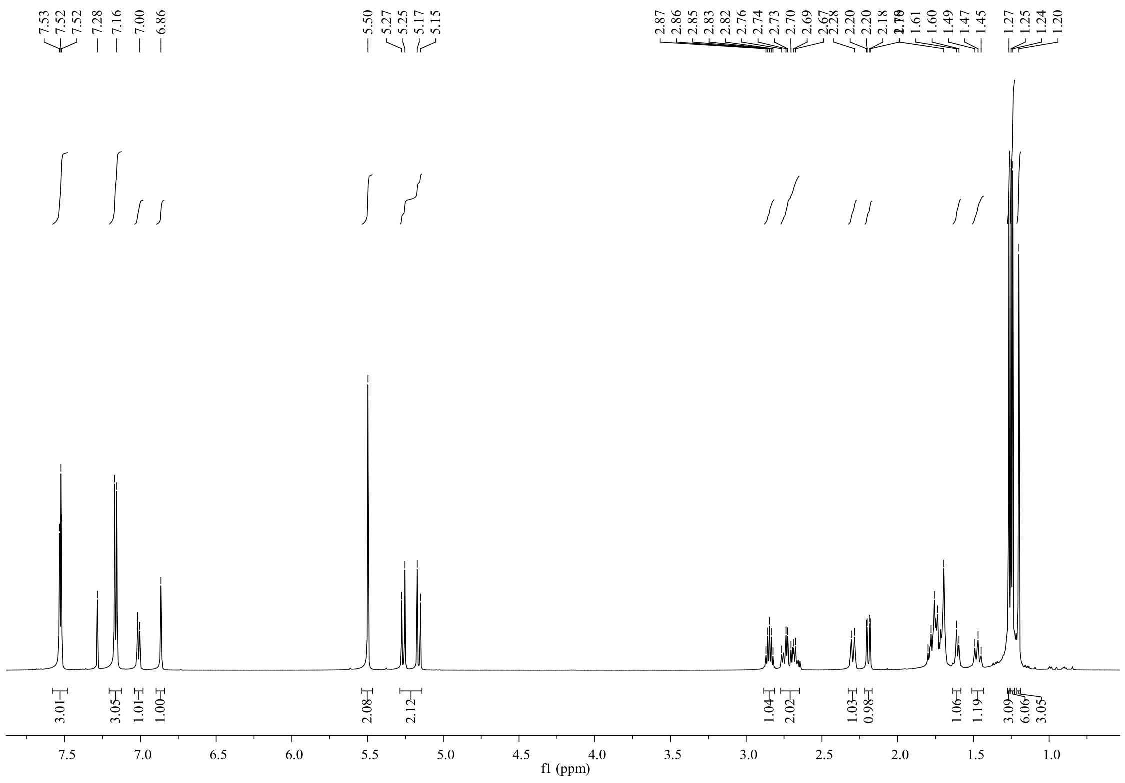

Figure S29. ^{1}H-NMR spectrum of **3j**

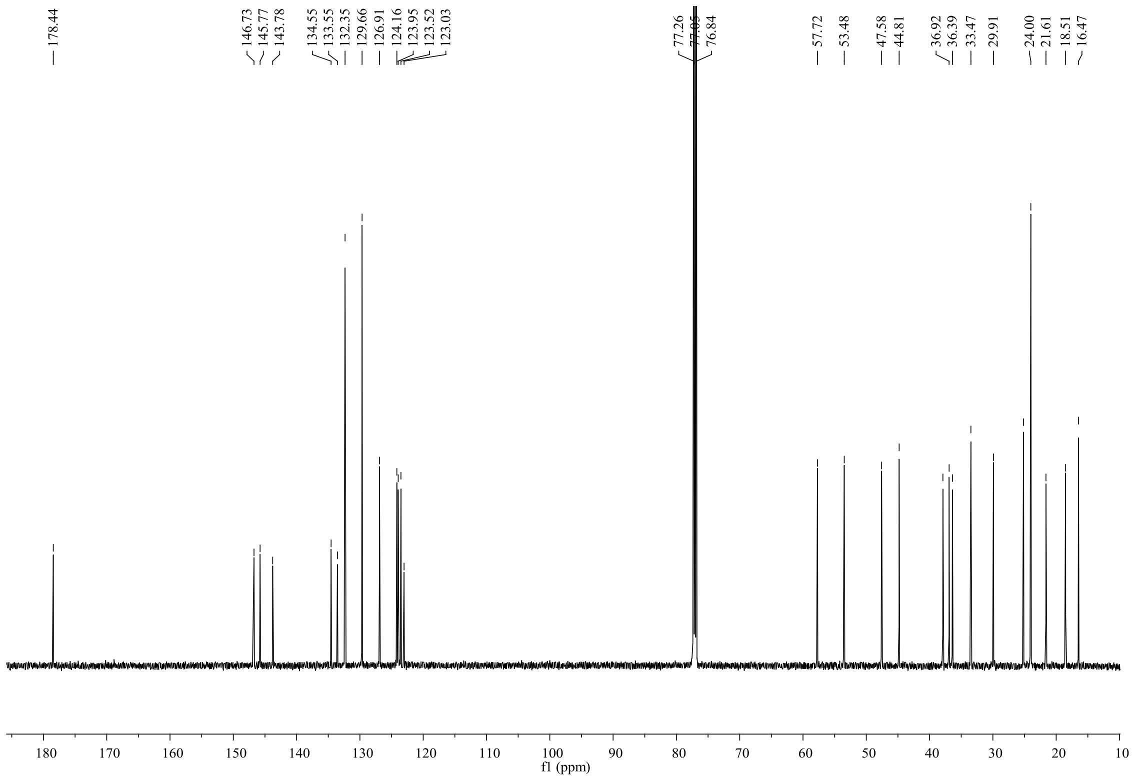

Figure S30. ^{13}C-NMR spectrum of **3j**

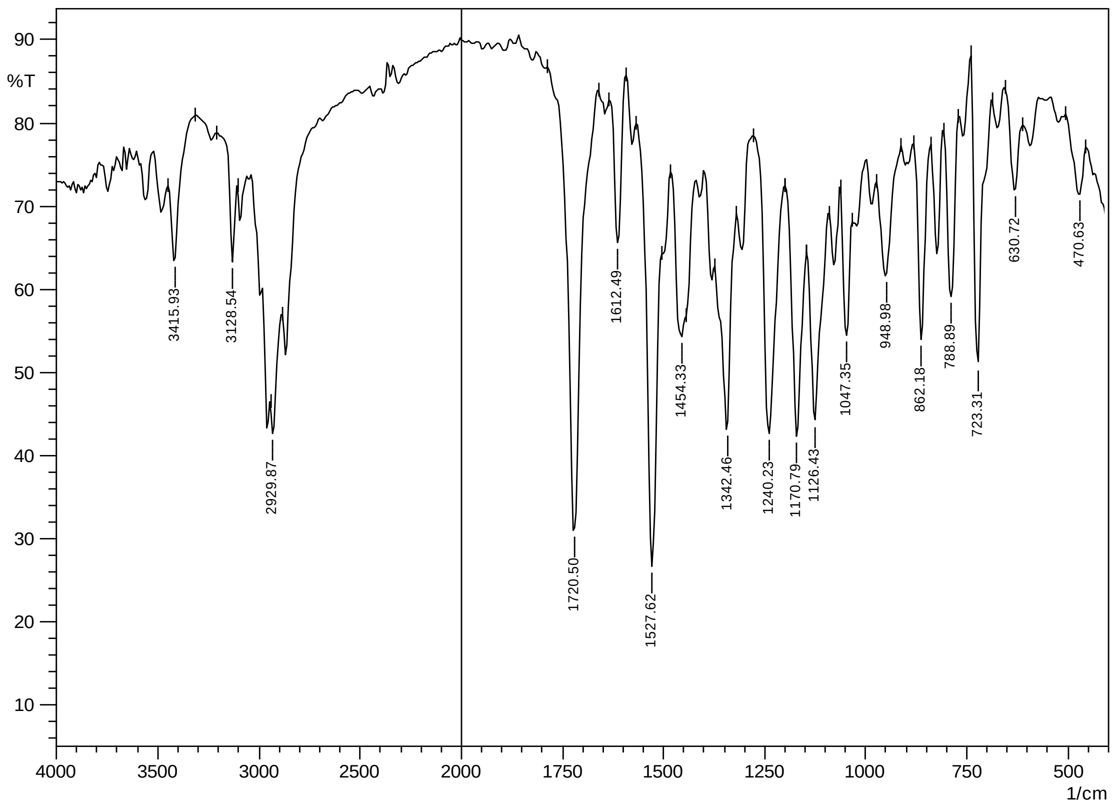

Figure S31. FTIR spectrum of **3k**

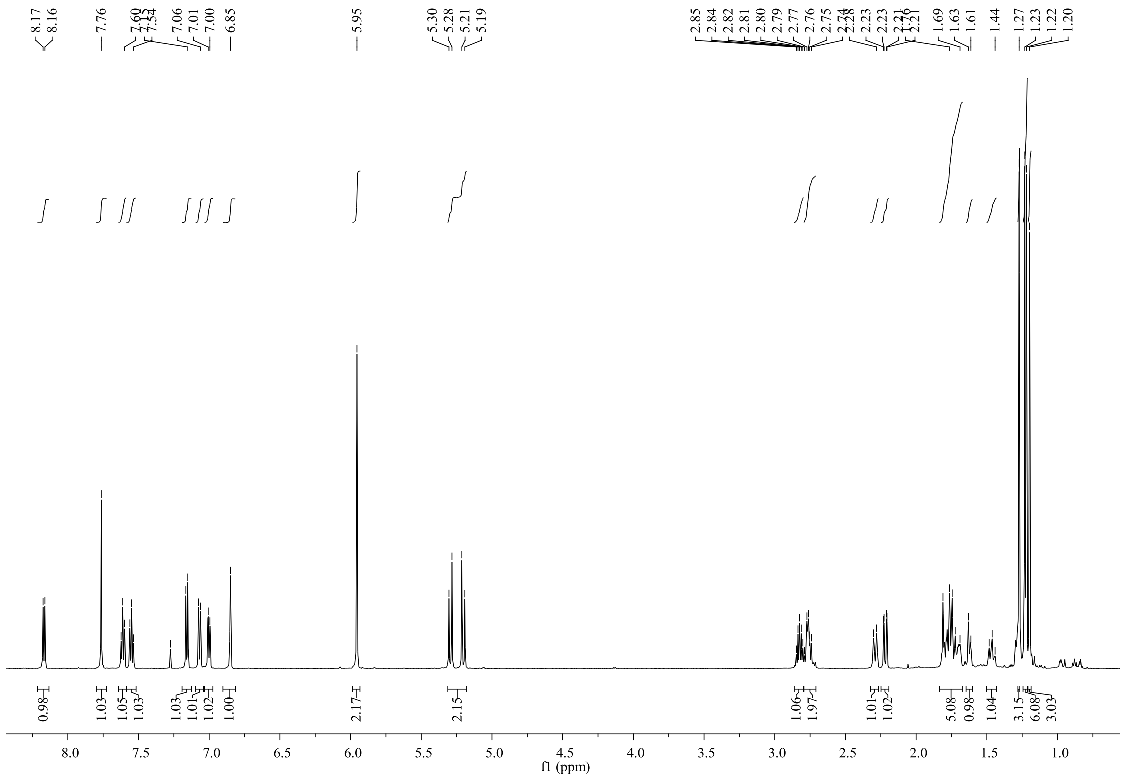

Figure S32. ^{1}H-NMR spectrum of **3k**

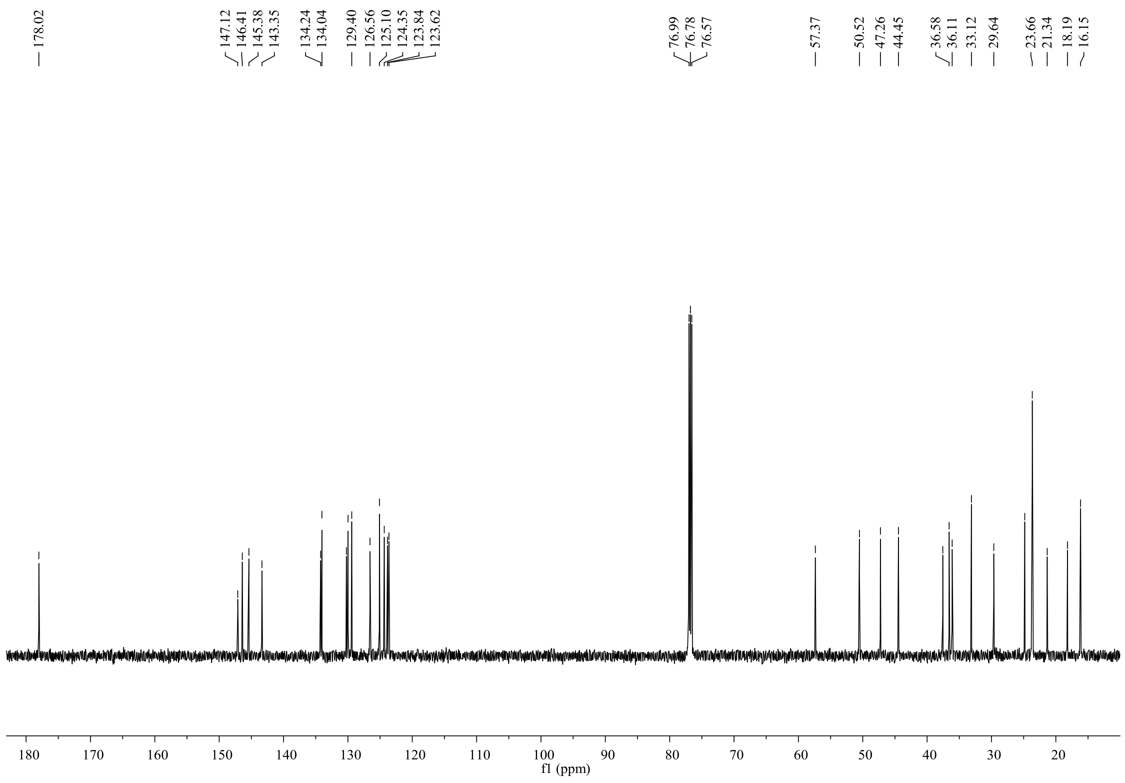

Figure S33. ^{13}C-NMR spectrum of **3k**

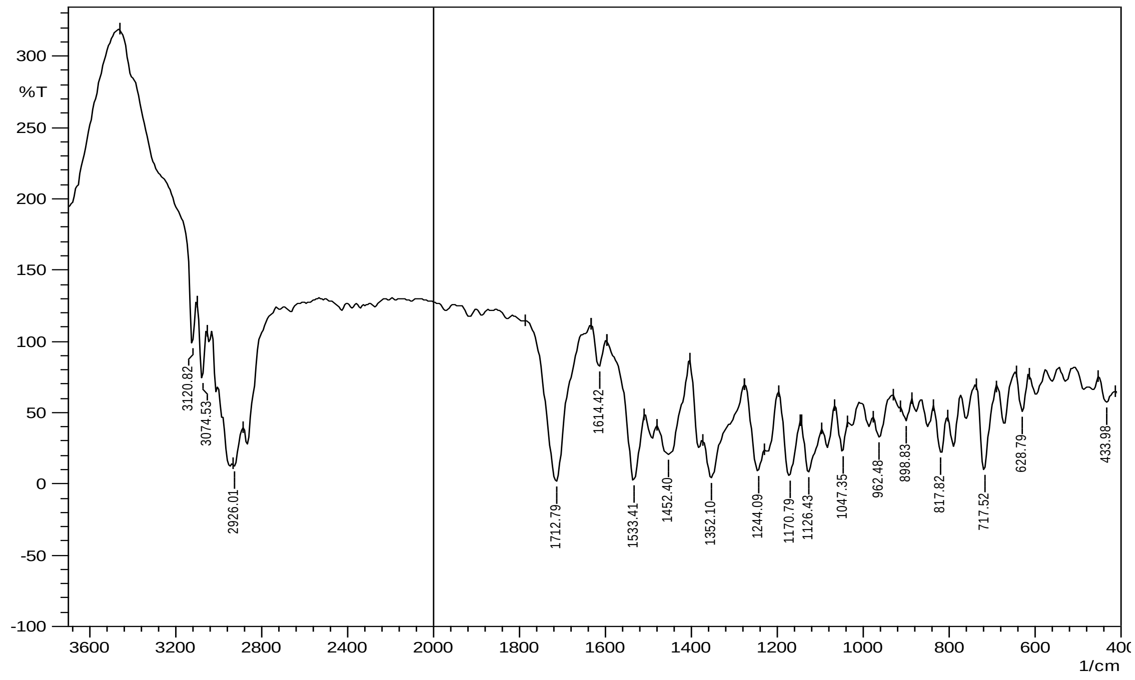

Figure S34. FTIR spectrum of **3l**

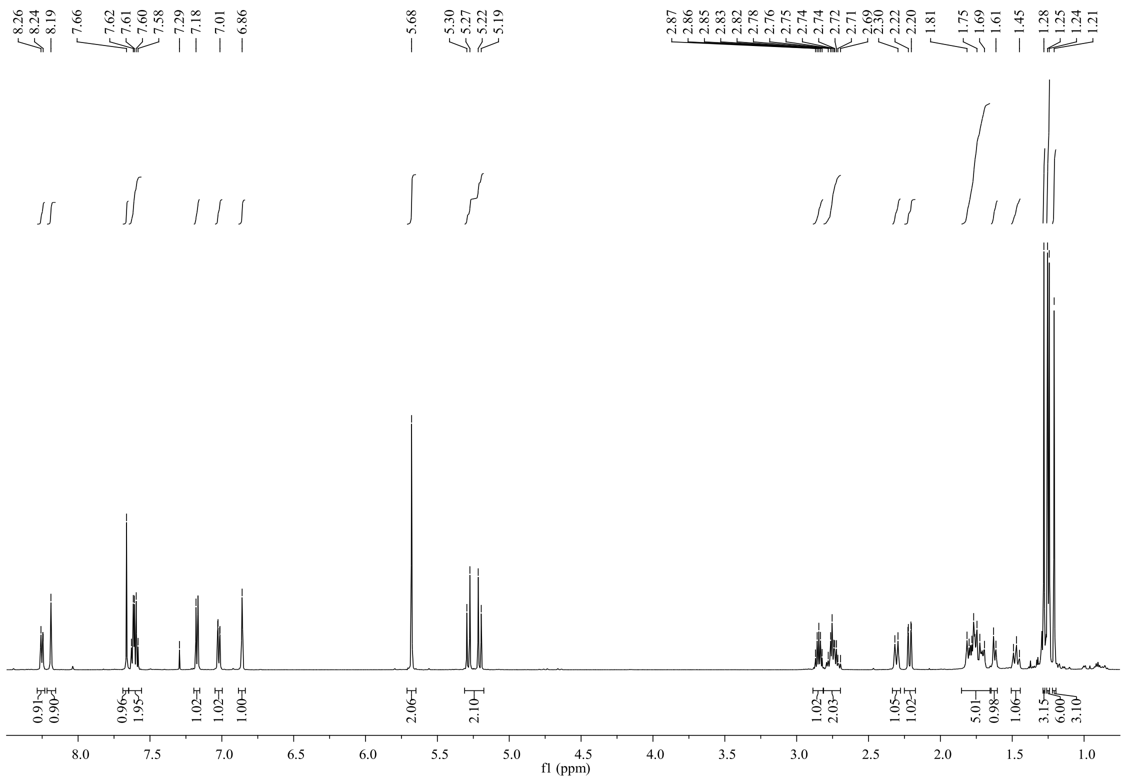

Figure S35. ^{1}H-NMR spectrum of **3l**

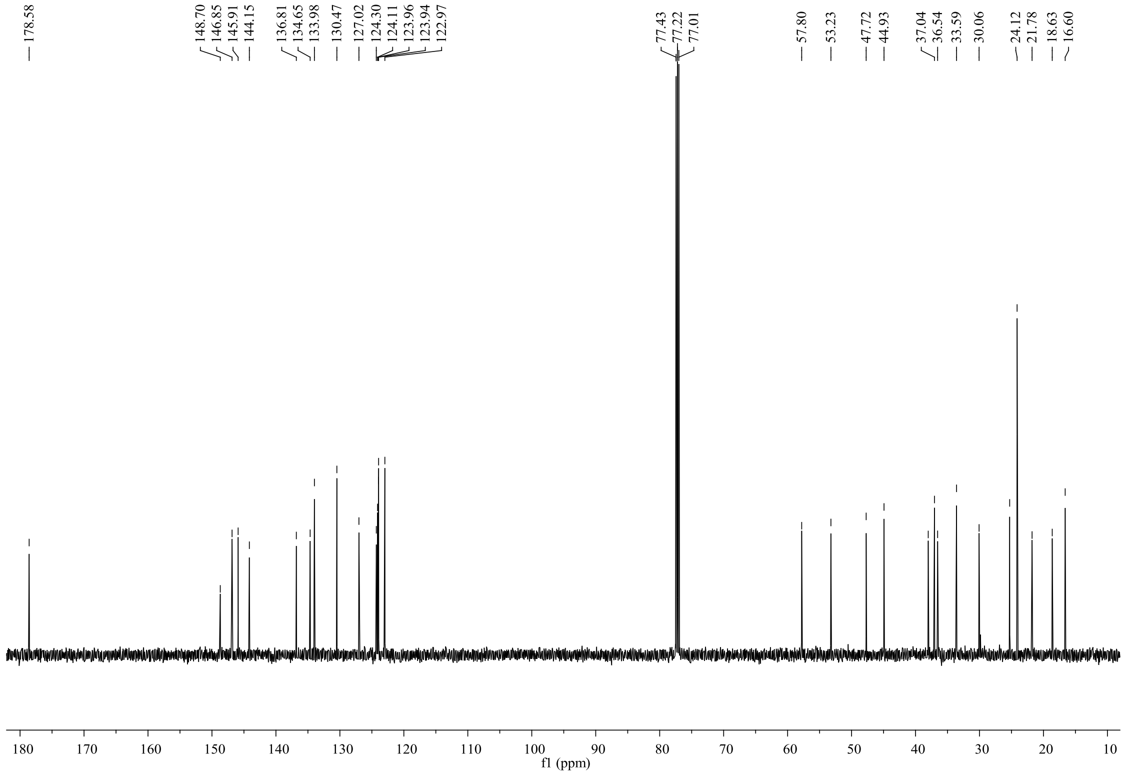

Figure S36. ^{13}C-NMR spectrum of **3l**

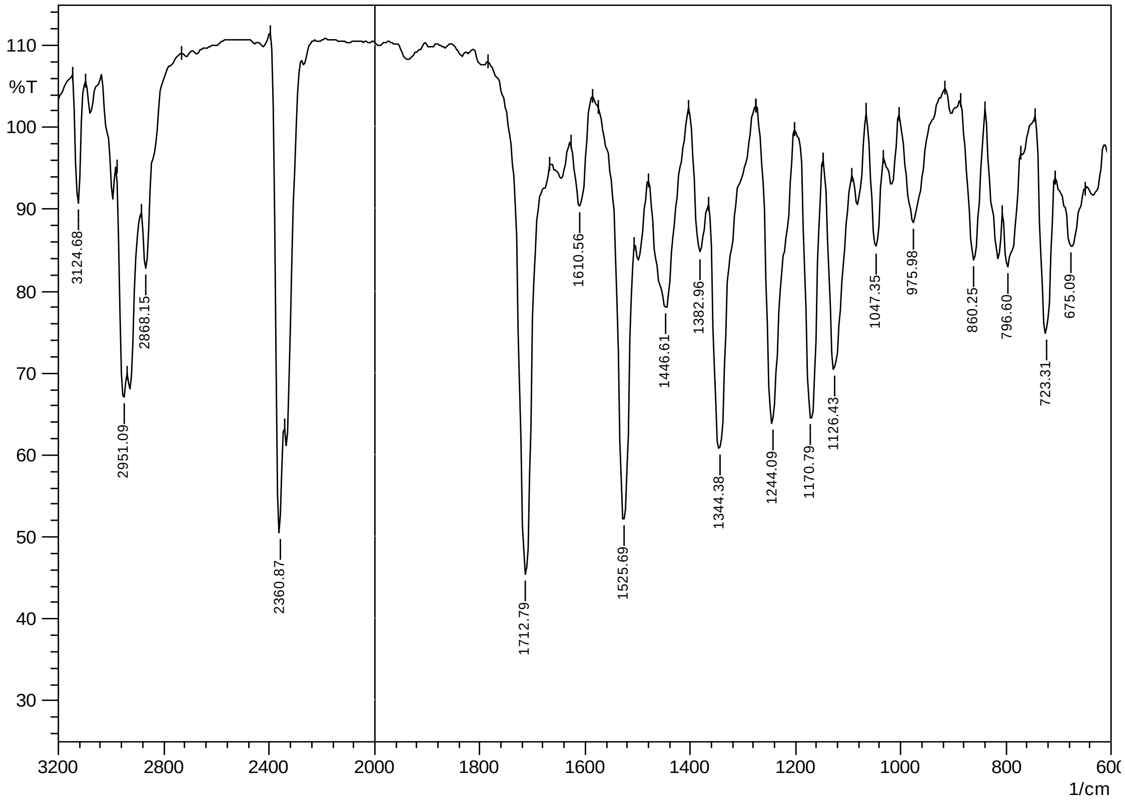

Figure S37. FTIR spectrum of **3m**

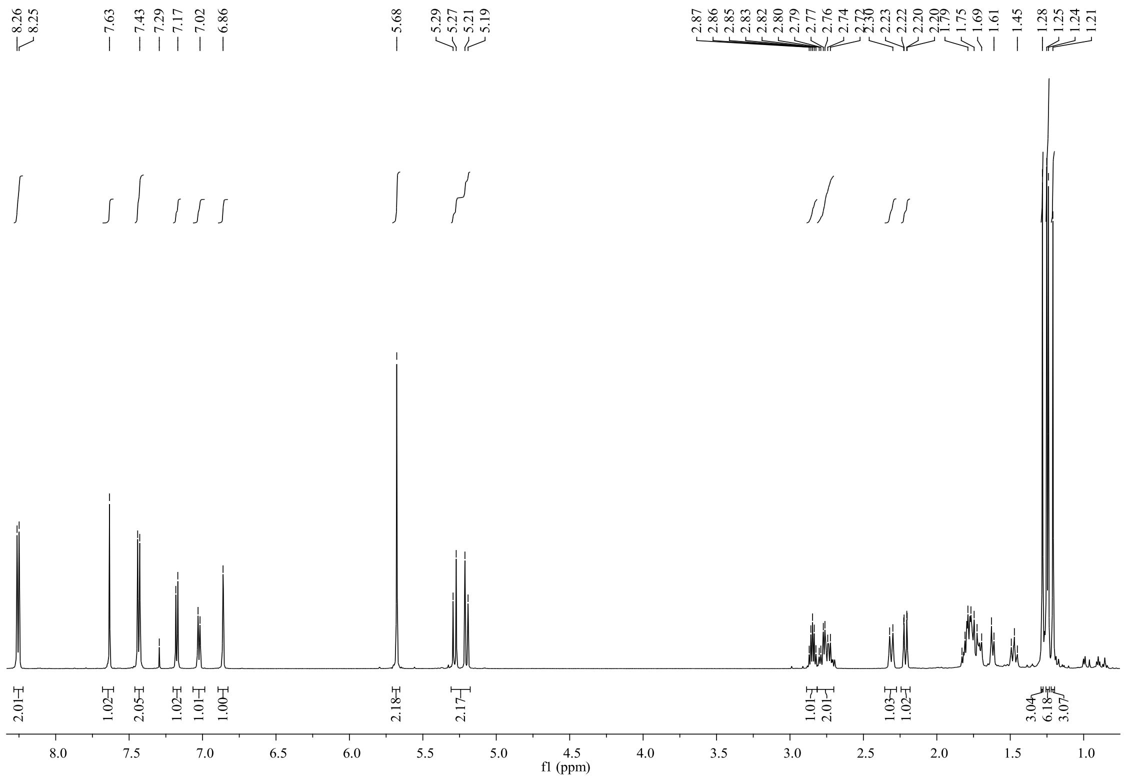

Figure S38. ^{1}H-NMR spectrum of **3m**

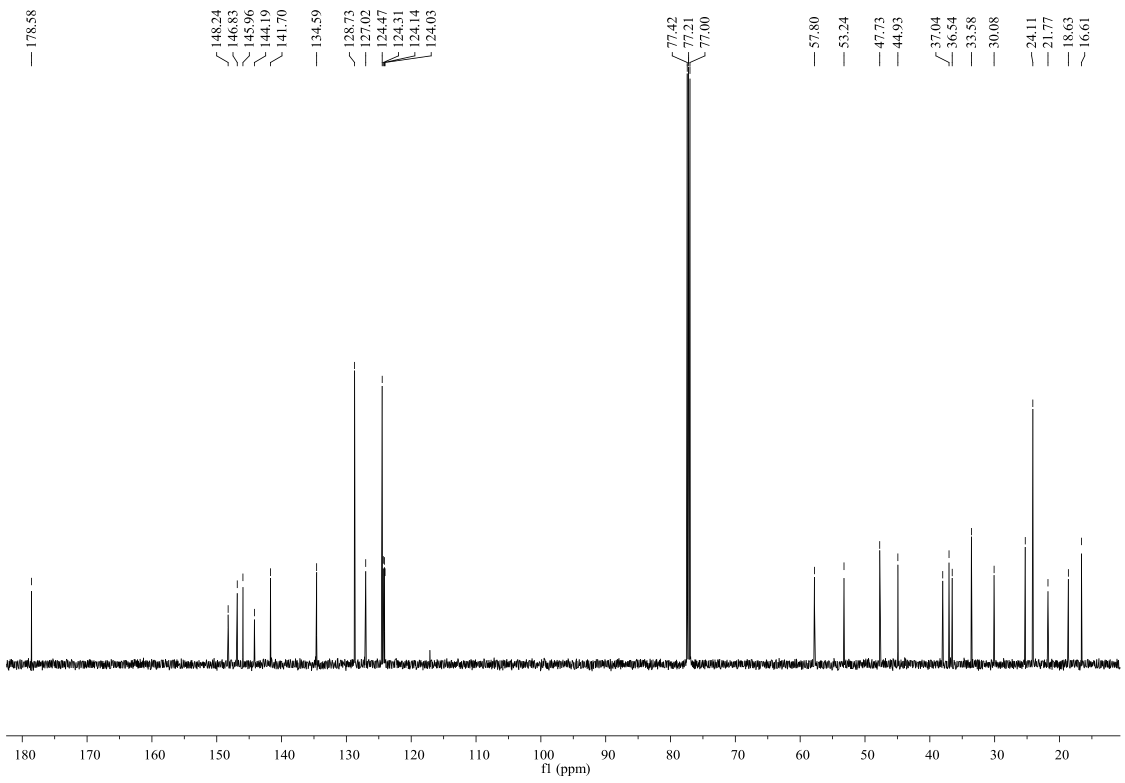

Figure S39. ^{13}C-NMR spectrum of **3m**

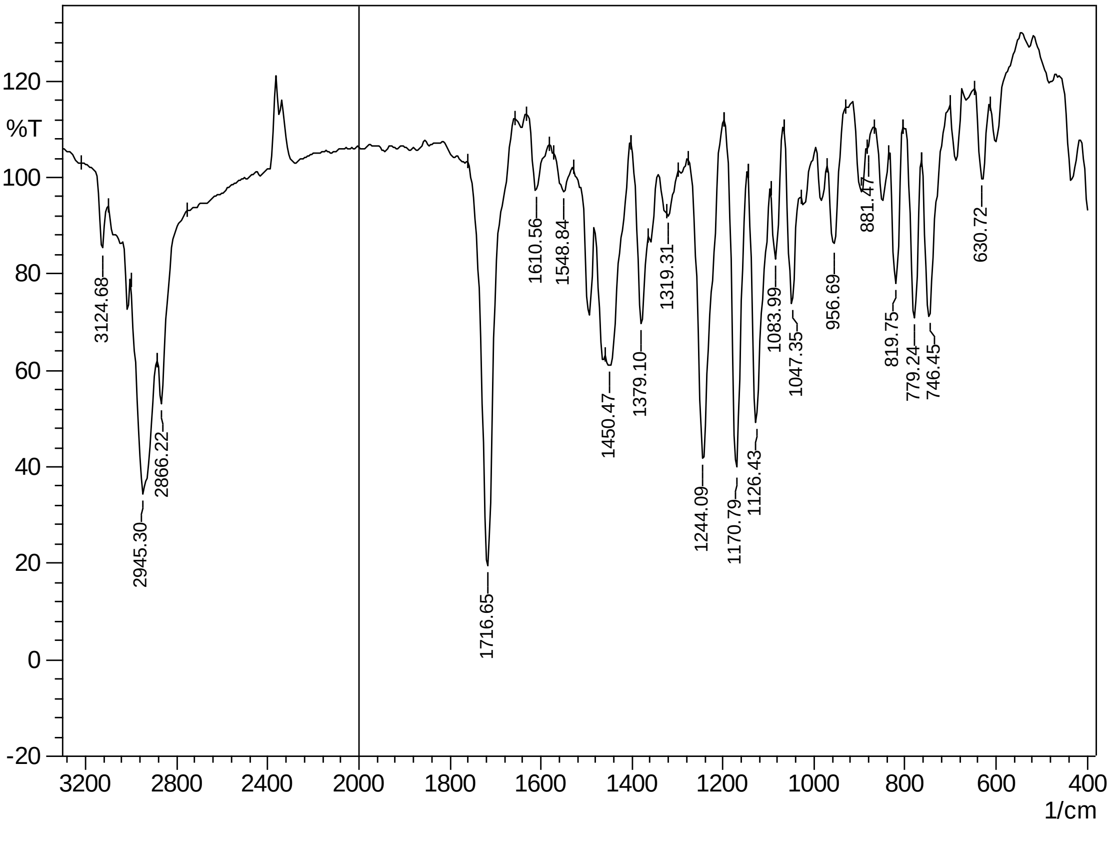

Figure S40. FTIR spectrum of **3n**

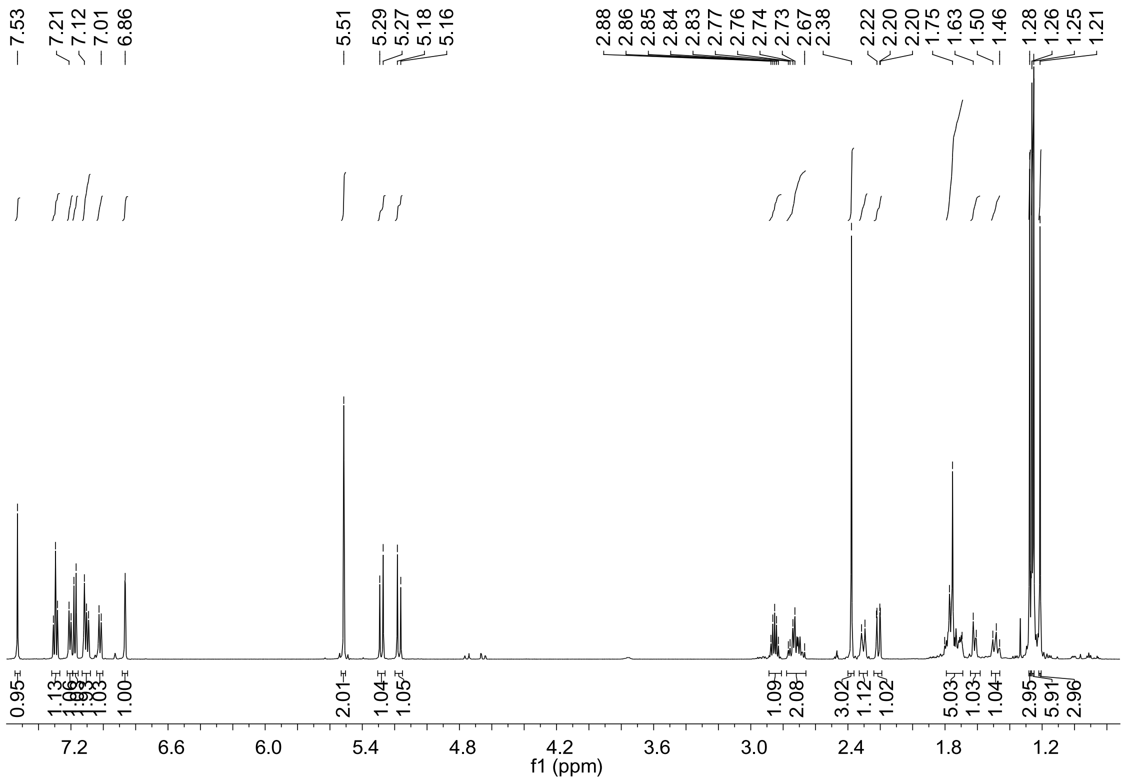

Figure S41. ^{1}H-NMR spectrum of **3n**

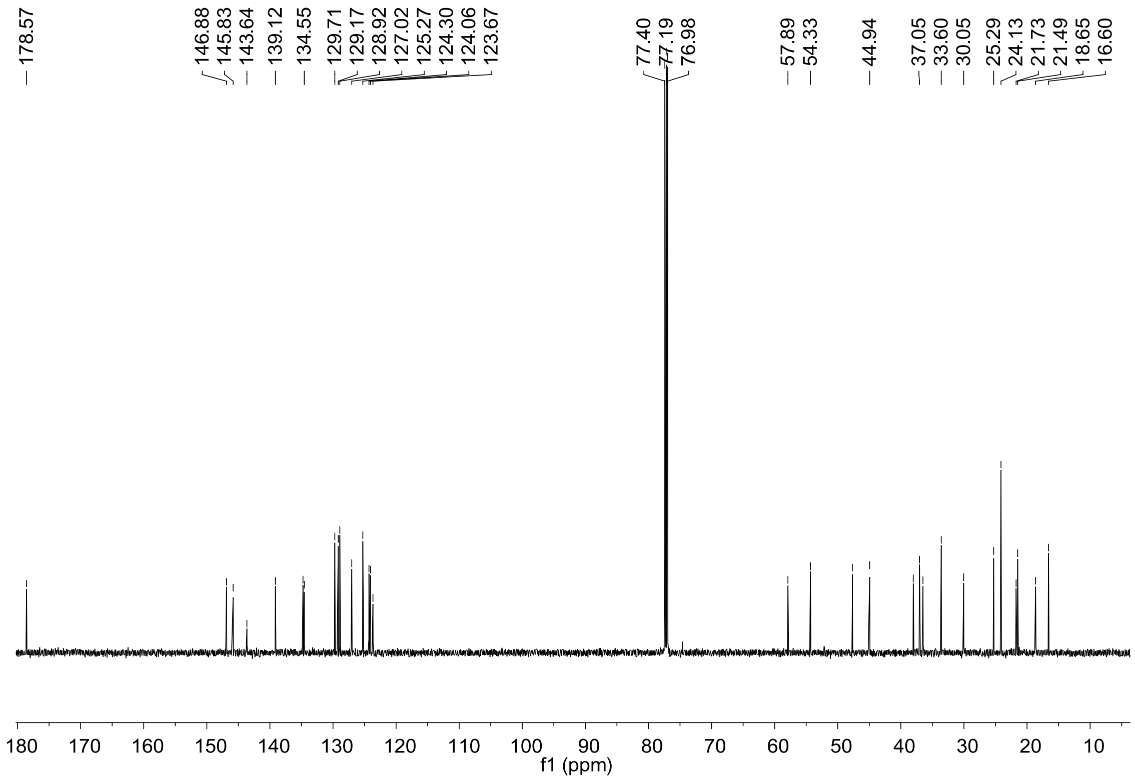

Figure S42. ^{13}C-NMR spectrum of **3n**

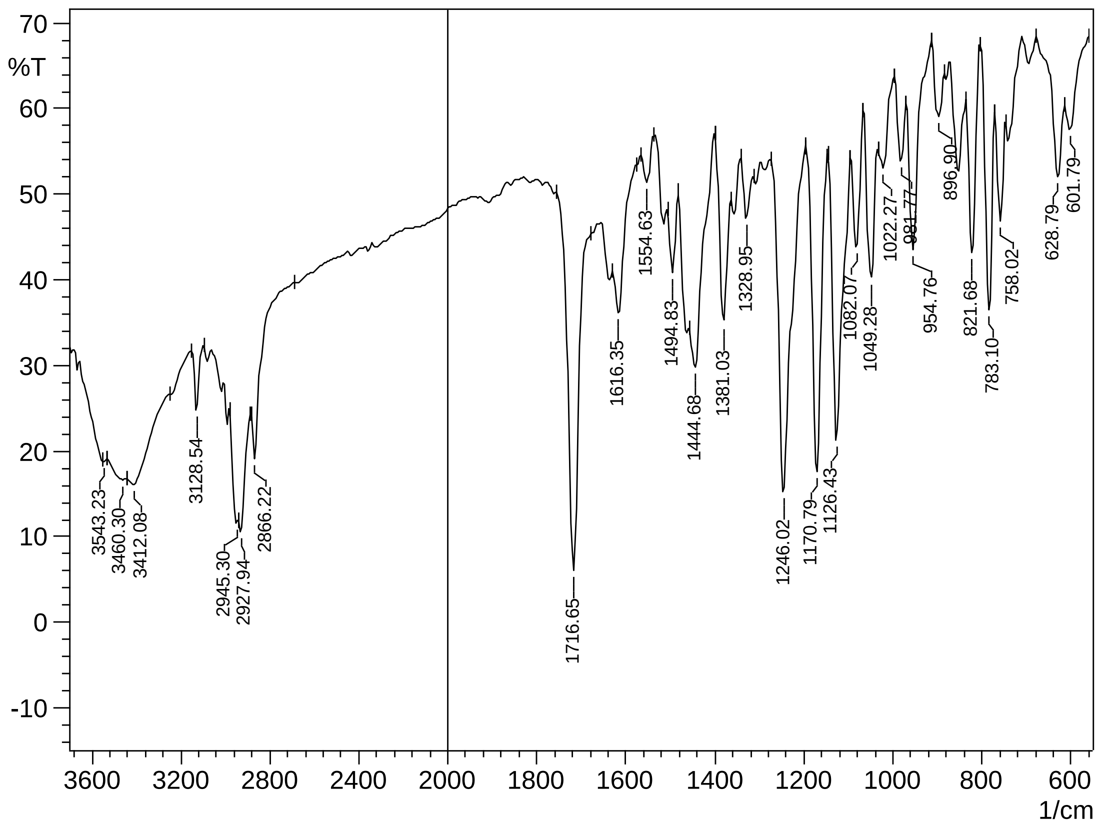

Figure S43. FTIR spectrum of **3o**

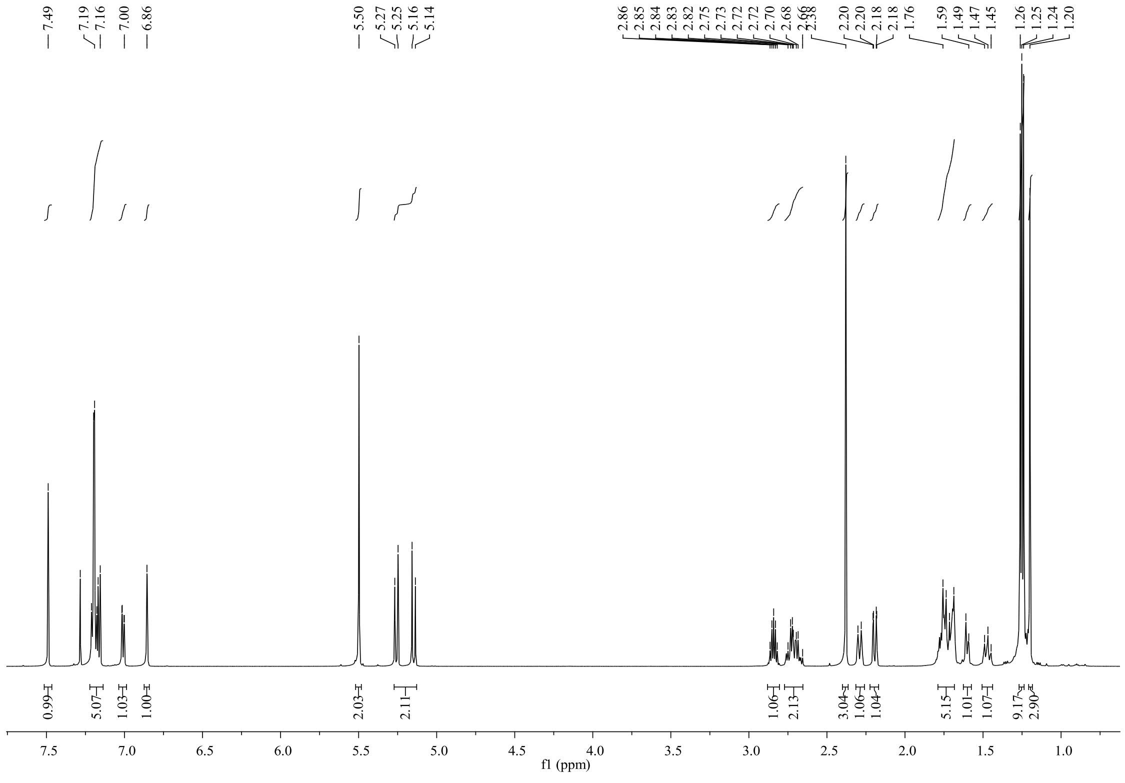

Figure S44. ^{1}H-NMR spectrum of **3o**

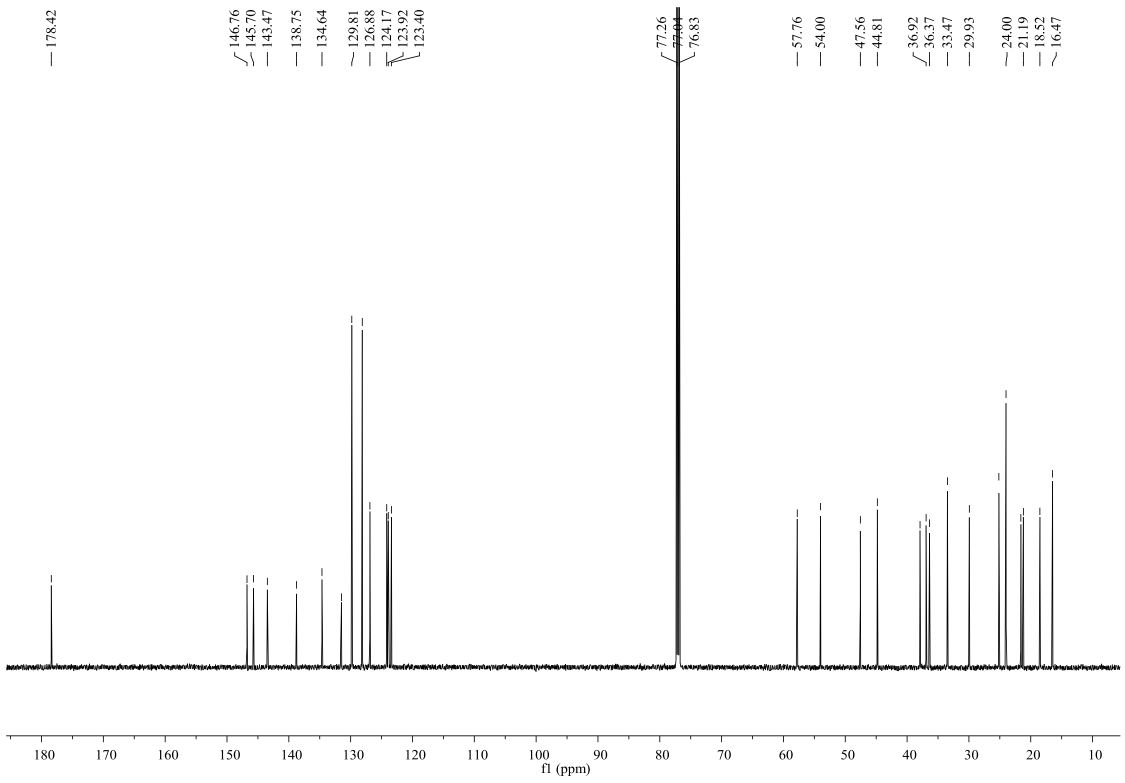

Figure S45. ^{13}C-NMR spectrum of **3o**

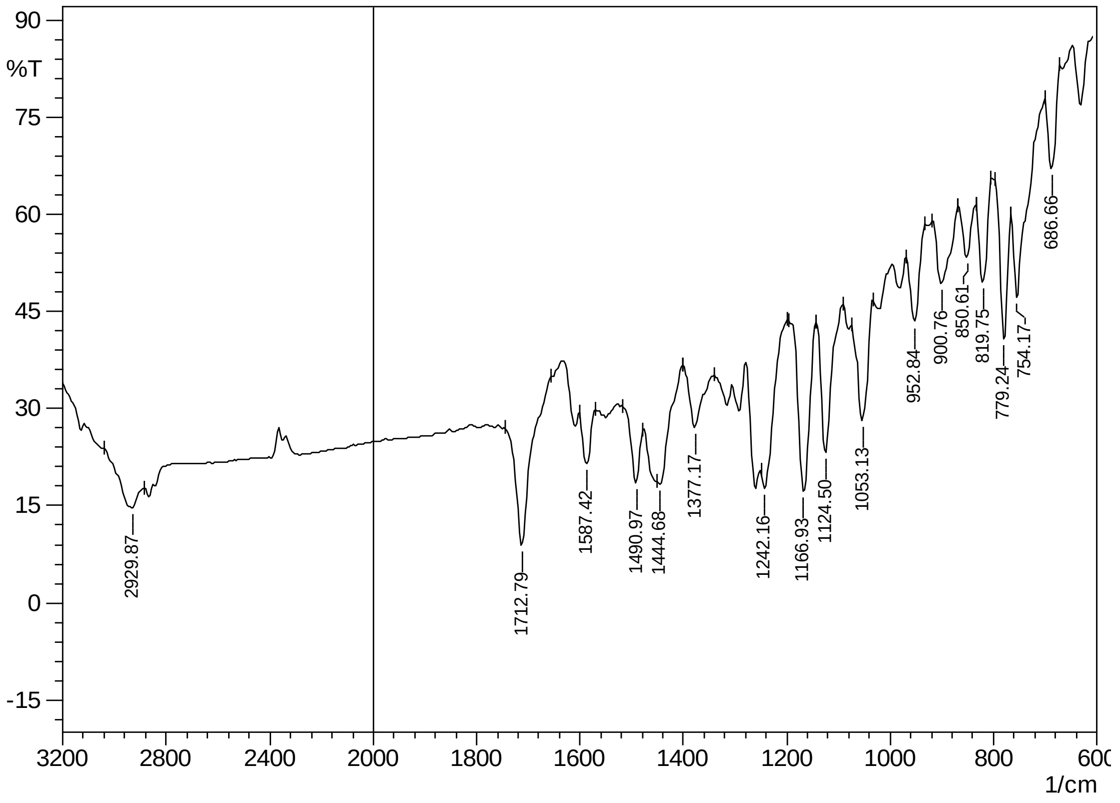

Figure S46. FTIR spectrum of **3p**

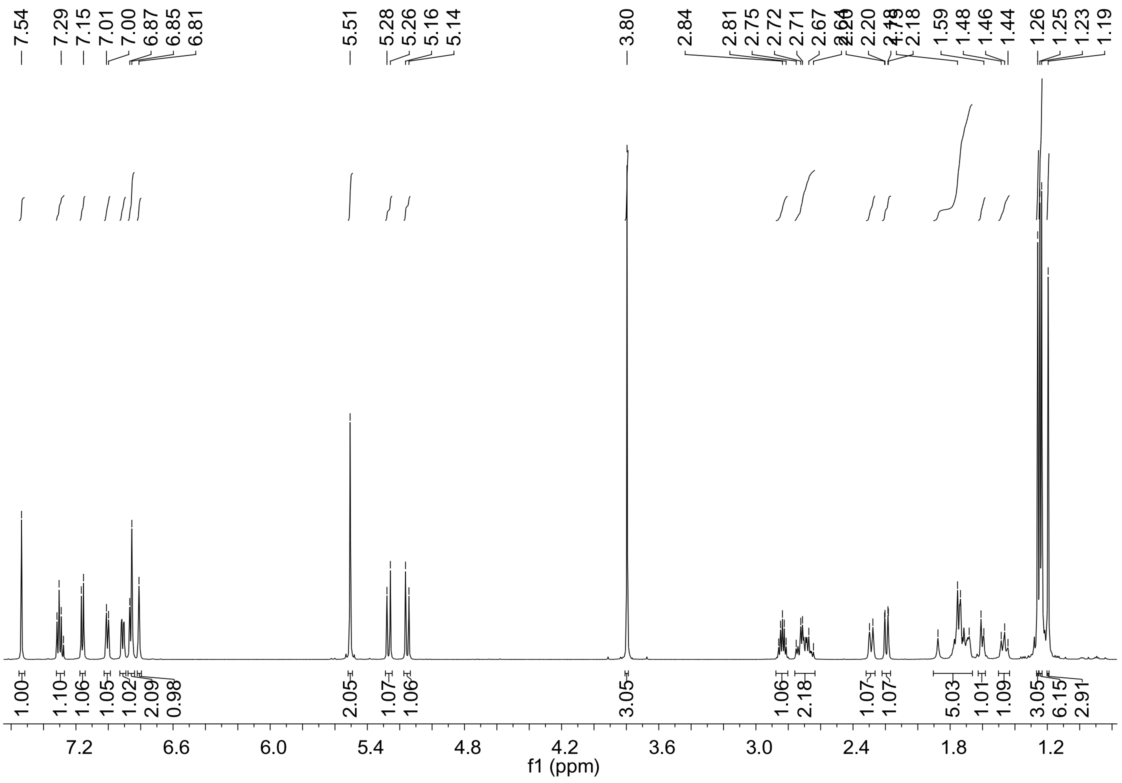

Figure S47. ^{1}H-NMR spectrum of **3p**

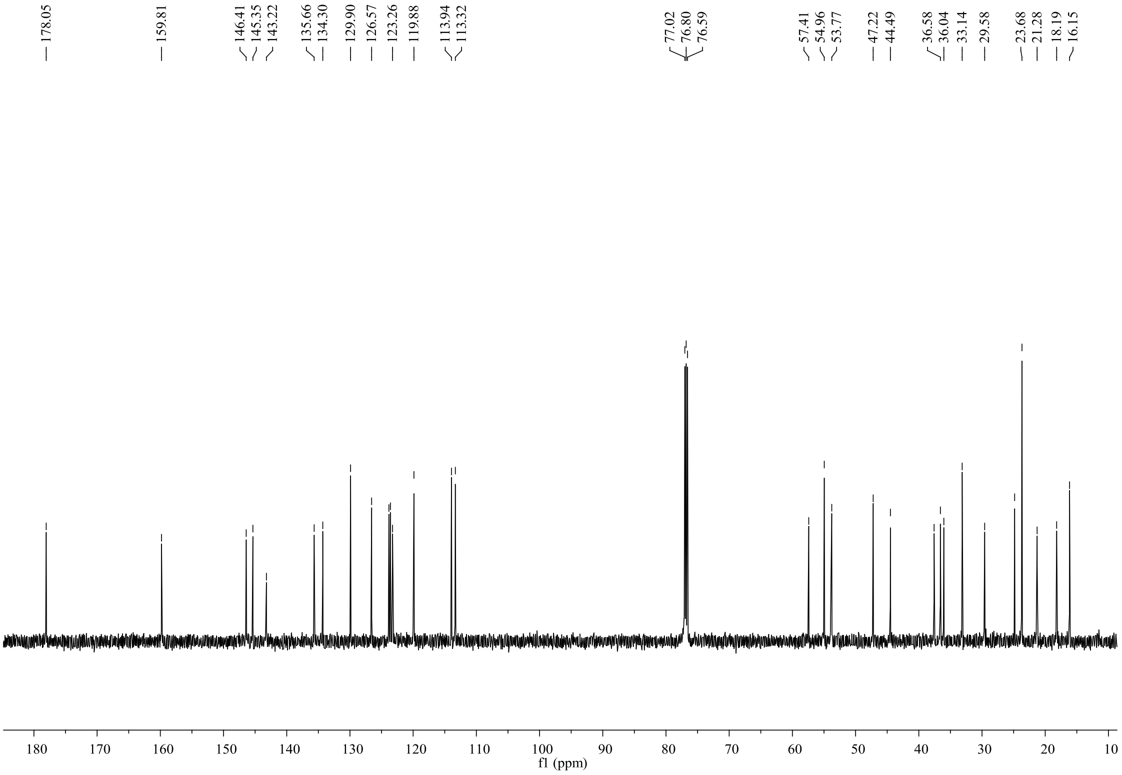

Figure S48. ^{13}C-NMR spectrum of **3p**
